# Violations of physical and psychological expectations in the human adult brain

**DOI:** 10.1162/imag_a_00068

**Published:** 2024-02-01

**Authors:** Shari Liu, Kirsten Lydic, Lingjie Mei, Rebecca Saxe

**Affiliations:** Department of Psychological and Brain Sciences, Johns Hopkins, Baltimore, MD, United States; Annenberg School for Communication, University of Pennsylvania, Philadelphia, PA, United States; Department of Computer Science, Princeton University, Princeton, NJ, United States; Department of Brain and Cognitive Sciences, Massachusetts Institute of Technology, Cambridge, MA, United States

**Keywords:** Social cognition, physical reasoning, cognitive development, prediction error

## Abstract

After seeing one solid object apparently passing through another, or a person taking the long route to a destination when a shortcut was available, human adults classify those events as surprising. When tested on these events in violation-of-expectation (VOE) experiments, infants look longer at the same outcomes, relative to similar but expected outcomes. What cognitive processes underlie these judgments from adults, and perhaps infants’ sustained attention to these events? As one approach to test this question, we used functional magnetic resonance imaging (fMRI) to scan the brains of human adults (total N = 49, 22 female, mean age of 26 years) while they viewed stimuli that were originally designed to test for physical and psychological expectations in infants. We examined non-mutually exclusive candidates for the processes underlying the VOE effect, including domain-general processes, like visual prediction error and curiosity, and domain-specific processes, like prediction error with respect to distinctively physical and psychological expectations (objects are solid; agents behave rationally). Early visual regions did not distinguish between expected and unexpected events from either domain. By contrast, multiple demand regions, involved in goal-directed attention, responded more to unexpected events in both domains, providing evidence for domain-general goal-directed attention as a mechanism for VOE. Left supramarginal gyrus (LSMG) was engaged during physical prediction and responded preferentially to unexpected events from the physical domain, providing evidence for domain-specific physical prediction error. Thus, in adult brains, violations of physical and psychological expectations involve domain-specific, and domain-general, though not purely visual, computations.

## 1 Introduction

In the first year of life, human infants rapidly develop expectations about the properties and behavior of inanimate objects, and animate agents. Like adults, they distinguish between surprising events and visually similar but unsurprising events (e.g. a ball rolls off the edge of a table, and hovers in midair, or stops rolling before it reaches the edge of the table). Infants look longer at the unexpected than expected outcome (the violation-of-expectation, or VOE, response) towards many events that adults rate as surprising (Shu et al., 2021; Smith et al., 2019): for example, when objects float in midair (Needham & Baillargeon, 1993) or appear to pass through each other (Spelke et al., 1992), and when agents change their goals (Woodward, 1998) or act inefficiently (Gergely & Csibra, 2003). For a recent meta-analysis over this literature, see Kunin et al. (2023). The mental processes that drive longer looking in these studies remain hotly debated (Aslin, 2007; Haith, 1998; Paulus, 2022; Stahl & Kibbe, 2022). Do infants respond to these events in virtue of domain-specific expectations about psychological and physical events (Baillargeon, 1995; Baillargeon et al., 2016)? Or are there stimulus-driven alternative explanations that could also explain these patterns of behavior (Bogartz et al., 2000; Rivera et al., 1999)? And does longer looking in infants reflect the detection of a surprising outcome, or also motivation to explore and explain the source of surprise (Sim & Xu, 2018; Stahl & Feigenson, 2019)?

### 1.1 Domain-specific hypotheses

One hypothesis regarding VOE effects in the developmental psychology literature is that surprising events violate *distinctively physical and psychological expectations*: that objects are solid and permanent; and that agents act efficiently towards goals. The strongest version of this hypothesis is that infants possess “core knowledge”: an early-emerging conceptual repertoire consisting of distinct systems for different domains of thought, including physics, psychology, number, and space (Carey, 2011; Spelke, 2022). There is evidence from developmental psychology that infants have distinct expectations for agents and objects: Infants represent objects as solid and permanent entities that do not hover in midair, or blip in and out of existence (Baillargeon, 1995). Infants represent agents as actors who have goals, and pursue them in consistent and efficient ways (Gergely & Csibra, 2003). There is also evidence that infants have some shared expectations across both domains. For example, infants expect that both agents and objects are solid entities (Saxe et al., 2006).

### 1.2 Domain-general hypotheses

Another broad hypothesis under consideration is that surprising events from violation-of-expectation studies evoke domain-general processes. One such process is *stimulus-driven prediction error* (i.e. a response to the visual features of the unexpected stimulus). While infant looking-time studies typically account for some simple perceptual alternative explanations, infants do reliably look longer at scenes that are visually novel (Frantz et al., 1962; Peeles & Teller, 1975). Furthermore, unexpected and expected events must be visually distinguishable, and thus each pair of events differs along at least one visual dimension. Developmental psychologists remain divided about whether for any pair of VOE stimuli, longer looking may be driven by distinctive visual features alone (Aslin, 2000; Haith, 1998).

A second domain-general hypothesis is that unexpected physical and psychological events evoke *curiosity and motivation* to gain information about the source of surprise (Sim & Xu, 2018; Stahl & Feigenson, 2019). Under this hypothesis, infant looking is not merely a passive behavior, but also an active process driven by the infant’s own learning goals (Raz & Saxe, 2020). There is some evidence that unexpected events evoke curiosity in infants. After viewing an unexpected physical event, such as a ball rolling through a solid wall, infants show enhanced learning about that object (Stahl & Feigenson, 2015), and choose to explore that object (Sim & Xu, 2017) as though they are trying to explain the outcome (e.g. by banging the ball after seeing a violation of solidity, and dropping the ball after seeing a violation of support; Stahl & Feigenson, 2015). In addition, infants only explore the object that violated their expectations when they have reason to be curious: They cease to explore if they receive an explanation for the surprising outcome (the wall has an archway through it, allowing the ball to pass through; Perez & Feigenson, 2022).

### 1.3 The contribution of functional neuroimaging for testing these hypotheses

Plausibly, all of these mental processes could influence infant looking, but which of these accounts for the VOE response? Despite decades of behavioral work, controversy remains. Here, we consider the potential contribution of neuroimaging to this debate, which can reveal the hidden internal processes underlying VOE by studying them simultaneously and directly.

If domain-specific processing underlies the VOE response, what brain regions could support those computations? In adults, different cortical regions represent the properties and dynamics of agents and objects. A set of regions including the temporoparietal junction (TPJ), medial prefrontal cortex (MPFC), precuneus (PC), and superior temporal sulcus (STS) are engaged during social perception and cognition (DiNicola et al., 2020; Koster-Hale et al., 2017). The STS, in particular, tracks other people’s actions, intentions, and interactions (Deen et al., 2015; Gao et al., 2012; Isik et al., 2017; Saxe, Xiao, et al., 2004; Shultz et al., 2011; Vander Wyk et al., 2009). A distinct set of regions including supplementary motor area, superior parietal cortex, and supramarginal gyrus (SMG), represents physical information including object mass and stability (Fischer et al., 2016; Pramod et al., 2022; Schwettmann et al., 2019). As early as has been measured, similar regions in infant brains are implicated in the processing of social versus physical stimuli (Farroni et al., 2013; Hyde et al., 2018; Lloyd-Fox et al., 2009; Wilcox et al., 2010), making studying these regions in adults relevant to hypotheses about the minds and brains of infants. Prior work measuring neural responses towards surprising physical and psychological stimuli has reported increased neural activity toward unexpected outcomes in regions associated with social and physical processing, as well as domain-general multiple demand (Marsh et al., 2014; Parris et al., 2009; Ramsey & Hamilton, 2010; Southgate et al., 2014), consistent with a neural prediction error (Friston, 2010): an increased response that encodes the difference between what was expected and what was observed. If these regions compute domain-specific prediction error in VOE events, then we expect to observe greater activity in each of these regions for unexpected events from the matching domain (e.g., a greater response to unexpected than expected physical events in SMG, and a greater response to unexpected than expected psychological events in STS).

By contrast, if early visual processing underlies the VOE response, then which regions would we expect to support this process? Early visual regions, including the primary visual cortex (V1) and middle temporal area (MT), are sensitive to a host of low-level visual features, including speed and direction of motion, and spatial extent, frequency, and orientation. New visual stimuli, relative to repeated visual stimuli, evoke activity in early visual regions, in both adults and infants (Emberson et al., 2015; Henson et al., 2000; Jiang et al., 2016). Thus, under the hypothesis that differences in stimulus features like visual orientation, motion, and frequency underlie the VOE response, we might expect to observe greater activity to unexpected than expected events, in both domains, in early visual regions, like V1 and MT.

If endogenous curiosity underlies the VOE response, a distinct set of regions would be recruited. Regions within the multiple demand (MD) network (Fedorenko et al., 2013), including regions in the frontal and parietal cortices, the insula, and the anterior cingulate cortex, respond with greater activity when human adults are engaged in a range of difficult versus easy tasks, regardless of the task’s modality (e.g., auditory vs. visual) or content (e.g., verbal arithmetic vs. motor inhibition). These regions are also engaged when people consider curiosity-inducing trivia questions (Kang et al., 2009), watch magic tricks (Parris et al., 2009), and learn from prediction error over rewards (Fouragnan et al., 2018). Studies of infants show similar effects: Regions along the lateral surface of the frontal and prefrontal cortices show greater activity to violations of a previously learned visual or auditory pattern (Ellis et al., 2021; Nakano et al., 2009; Werchan et al., 2016). Thus, if domain-general endogenous attention underlies the VOE response, then we would expect regions in the multiple demand network to respond with greater amplitude to unexpected than expected events from both domains.

**Fig. 1.**
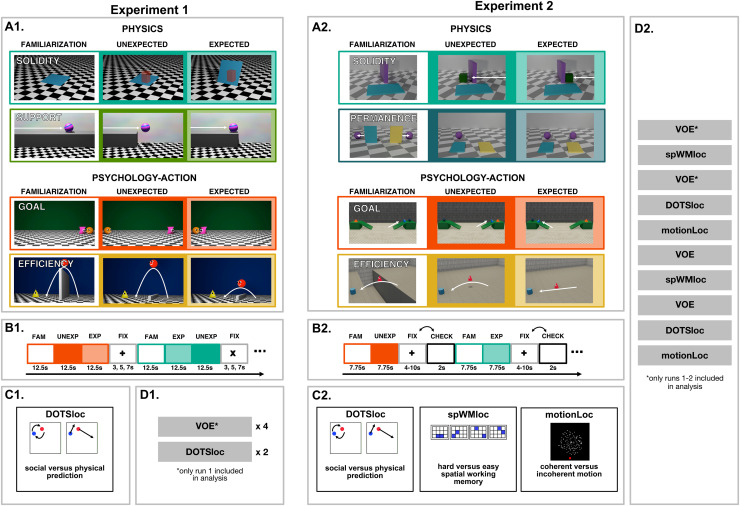
Overview of the methods of Experiments 1-2. (A1-2) Overview of VOE task. (top half) Stimuli from the domain of physics, including violations of object *solidity* and *permanence*. (bottom half) Stimuli from the domain of psychology, where the source of the violation is the agent performing a surprising action (psychology-action, including violations of *goal*-directed action and action *efficiency*). See Figure 6 for stimuli involving surprising physical outcomes, revealed by an agent’s action, that were also included in Experiment 2. (B1-2) Structure of VOE run, with each trial containing a familiarization movie followed by both an expected and unexpected movie (Experiment 1), or an expected or unexpected movie (Experiment 2). (C1-2) Localizer tasks and contrasts for physics and psychology regions (interacting dots localizer, DOTSloc), multiple demand regions (spatial working memory localizer, spWMloc), and area MT (motionLoc). (D1-2) Sequence of functional runs, which occurred after anatomical scans.

### 1.4 Overview of current research

**Fig. 2.**
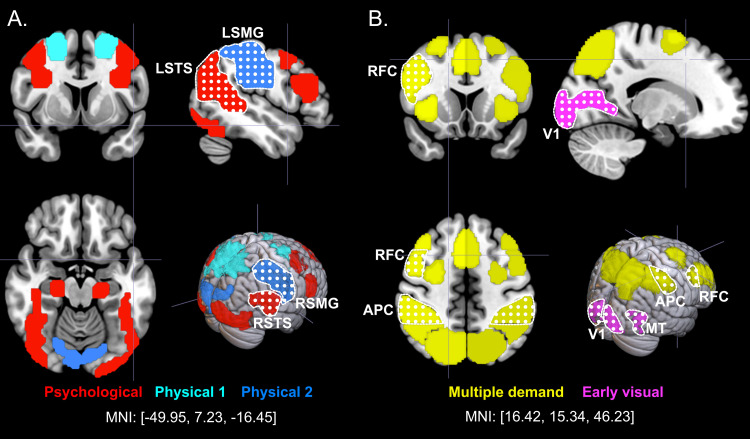
(A) Domain-specific and (B) domain-general parcels from Experiments 1-2, overlaid on an MNI152 template brain. Focal regions are indicated using the dotted pattern; these regions were pre-registered in Experiment 2, including left and right supramarginal gyrus (LSMG, RSMG), left and right superior temporal sulcus (LSTS, RSTS), right frontal cortex (RFC), anterior parietal cortex (APC), primary visual cortex (V1), and middle temporal area (MT). (A) The full set of domain-specific regions we explored, including frontoparietal parietal regions implicated in physical understanding, and frontal regions implicated in action observation. (B) The full set of domain-general regions we explored, including more multiple demand regions. These two broader sets of regions were pre-registered in Experiment 2. MNI coordinates identifying the X, Y, and Z slice positions are listed below each figure. All data used to make these parcels were independent of the data used to extract responses in the primary VOE task in both experiments (see Section 2.6.1 and SM for details about parcel definition).

Here, we sought complementary evidence to the debate about infant VOE effects, by scanning the brains of adults while they watched events that were designed to test for physical and psychological expectations in infants. We studied cortical regions likely to be involved in the hypothesized processes underlying the VOE response (psychological and physical prediction, early visual processing, and goal-directed attention; see Fig. 2) in subject-specific functional regions of interest (ssfROIs), defined using validated localizer tasks from prior literature (Fedorenko et al., 2013; Fischer et al., 2016; Robertson et al., 2014). We then measured the responses of these regions to unexpected and expected psychological and physical events designed for infant studies. We tested whether the responses in each region are driven by manipulations of domain (psychology vs. physics), event type (expected vs. unexpected), or an interaction of these factors. Under domain-specific hypotheses, we expect an interaction between domain and event, with putative physics regions responding more to unexpected than expected physical events, but not psychological events, and vice versa for putative psychological regions. Under domain-general hypotheses, we expect greater responses to unexpected events for both domains in early visual or multiple demand regions.

Our approach has both strengths and weaknesses. Studying adult brains, rather than infant brains, allows us to identify regions involved in each hypothesized process in individual participants using independently validated localizer tasks. This procedure gives us more confidence that the responses we measure correspond to the hypothesized mental processes, strengthening our reverse inference from neural activity to cognitive mechanisms (Fedorenko, 2021; Poldrack, 2006). Since there is a strong correspondence between the large-scale topography of adult brain networks between adults and infants, as early as they can be measured (Eyre et al., 2021; Kosakowski, Cohen, Takahashi, et al., 2021), insights from adult brains could directly guide future studies of infant brains. However, researchers remain divided on how much continuity there is between infant and adult brains (Blumberg & Adolph, 2023; Liu et al., 2023). We will return to the strengths and weaknesses of our approach in the discussion.

## 2 Methods

Here, we present the methods of two pre-registered experiments. We first conducted Experiment 1 and pre-registered whole-brain random effects analyses, which led to no consistent violation-of-expectations results across all runs and participants (see Supplementary Material [SM], Section 8). We then conducted a series of exploratory region-of-interest (ROI) analyses over the same dataset, which led to potentially interesting results. In order to evaluate the robustness of these results, we conducted a second pre-registered experiment, Experiment 2, on a larger sample of participants, with more stimuli and localizer tasks. Because the experiments and their results are similar, we report the methods and results folding across experiments. Conducting two experiments allows us to evaluate the robustness of our findings; thus, we will make the strongest claims about findings that replicate in both samples, generalizing across stimulus materials and design choices.

### 2.1 Open science practices

The methods and analyses of both experiments were pre-registered prior to data collection, including several updates. Our pre-registration documents, openly available at https://osf.io/sa7jy/registrations, detail all decisions and updates for both experiments. All experiment scripts, including stimuli shown to participants, as well as the data and analysis scripts required to reproduce statistical results, can be found at https://osf.io/sa7jy/. De-faced brain images from participants in Experiments 1 and 2 who consented to share them (N = 16/17 for Experiment 1; N = 29/32 for Experiment 2) can be found on OpenNeuro (https://openneuro.org/datasets/ds004934).

### 2.2 Participants

We recruited 20 participants (Mean age = 25.1 y, range = 19-45; 17 right-handed; 15 female, 5 male; 10 White; 10 Black, Asian, or Latine) for Experiment 1, and 33 participants (Mean age = 25.7 y, range 18-45; 30 right-handed; 21 female, 12 male; 19 White; 14 Black, Asian, Latine, or multiracial) for Experiment 2, all from the Boston area. Two participants were excluded from Experiment 1 due to technical issues. One participant each was excluded from Experiment 1 and Experiment 2 for not contributing usable, low-motion fMRI data. This left a final sample of N = 17 for Experiment 1, and N = 32 for Experiment 2. Participants had normal or corrected-to-normal vision and no MRI contraindications. We chose the sample size for Experiment 2 using a combination of simulation power analyses over Experiment 1 (see pre-registration for details), and other considerations of time and cost. All study procedures were approved by the MIT Committee on the Use of Humans as Experimental Subjects. Participants were asked to provide written informed consent before participation, and were paid $30 per hour.

### 2.3 Experimental tasks

**Fig. 3.**
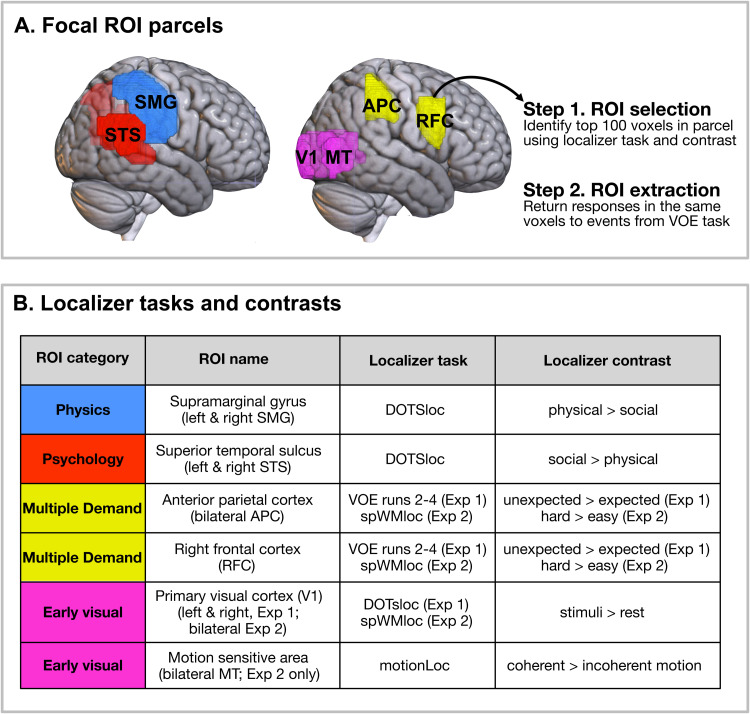
(A) Parcels or search spaces for all focal regions of interest (ROIs) in Experiments 1-2. (B) Localizer tasks and contrasts for subject-specific functional ROI selection in Experiments 1-2. Information specific to each experiment is indicated explicitly (e.g., bilateral MT was an ROI in Experiment 2 only; in the absence of a localizer for the multiple demand network in Experiment 1, independent data from the VOE task were used to identify the multiple demand ROIs).

See Figures 1C and 3B for an overview of our localizer tasks, and Figures 1A-1B for an overview of our primary VOE task. In both experiments, localizer tasks were used to identify regions of interest in individual participants; these ROIs were then used to study the responses in the primary VOE task. Localizer tasks have been validated in prior literature; we conducted an additional validation analysis for these tasks, which we report in the SM.

#### 2.3.1 Localizer task, Experiment 1

The DOTSloc task (Fischer et al., 2016) reliably evokes responses in the superior temporal sulcus (STS) and supramarginal gyrus (SMG) (ROIs for psychological and physical prediction). Stimuli consisted of 32 unique 10-s movies of two dots moving as though they are physical objects, or as though they are interacting socially. Participants watched the dots, imagined the trajectory of one of the dots when it disappeared briefly, and had 1.5-s to indicate whether the final position of the hidden dot matches what they imagined using a button press. Each run included 19 26-s blocks (two 10-s videos and 1.5-s response periods per block; 8 physical blocks, 8 social blocks, and 3 rest blocks). On social and physical blocks, participants saw two different videos from the corresponding condition. Participants saw two runs, each of which lasted approximately 8.2 minutes.

#### 2.3.2 Primary VOE task, Experiment 1

Our violation-of-expectation (VOE) task (Fig. 1, panel A1) from Experiment 1 consisted of 4 handcrafted sets (“scenarios”) of animated videos, adapted directly from previous studies from the infant cognition literature, involving violations of goal-directed action (*goal*) (Woodward, 1998), action efficiency (*efficiency*) (Gergely et al., 1995), object solidity (*solidity*) (Baillargeon et al., 1985), and object support (*support*) (Needham & Baillargeon, 1993).

Each VOE run had an event-related design (Fig. 1, panel B1): 8 trials (2 apiece of the solidity, support, goal, and efficiency scenarios), with jittered fixation/attention check periods of 3, 5, or 7 s in between each trial, and then a final rest period. Each trial had 3 parts: a familiarization movie followed by two test movies, expected and unexpected, presented in random order. All movies lasted 6 s with a 250 ms interstimulus interval, and each movie played twice in a row each time it was presented, followed by a jittered fixation/attention check. Participants were asked simply to pay attention to the movies. During the fixation period, participants pressed a button if the fixation cross was the letter X instead of a plus symbol (+) (33% of trials). The stimuli flipped horizontally for half of the trials to introduce minor visual variability across the run. All participants saw four runs, each of which lasted approximately 8.0 minutes.

#### 2.3.3 Localizer tasks, Experiment 2

Experiment 2 included the DOTSloc task, as well as two additional localizer tasks (Fig. 1, panels B2-C2). Like in Experiment 1, the DOTSloc task was used to define ROIs for STS and SMG, and all but two participants saw two runs of this task. The remaining two participants only underwent one run due to time restrictions.

The spWMloc task (Fedorenko et al., 2013), openly available at https://evlab.mit.edu/funcloc/, identifies regions in the multiple demand (MD) network, including bilateral anterior parietal cortex, and right frontal cortex (ROIs for goal-driven attention). This task was also used to identify primary visual cortex (V1). Stimuli were rectangular 8-by-8 grids. Participants saw a sequence of grid-squares change color, either one (easy condition) or two (hard condition) at a time. They were asked to remember the locations of the changing squares over the sequence, and indicated using a button press which of two alternative grids matched the resulting layout, with feedback. Participants saw two runs, except for one participant who only underwent one run due to time restrictions. Each run included 20 16-s blocks (6 easy, 6 hard, and 4 rest blocks), and lasted approximately 7.5 minutes.

The motionLoc task (Robertson et al., 2014) identifies motion-sensitive middle temporal area (MT) (ROI for early visual processing). Participants fixated on a red dot near the bottom center of the screen while a large circular space of small moving dots played above fixation. The dots moved either coherently (in a uniform direction) or randomly around the space. Participants pressed a button whenever the red dot flickered. Participants saw two runs, except for two participants who only underwent one run due to time restrictions. Each run lasted approximately 4.6 minutes.

#### 2.3.4 Primary VOE task, Experiment 2

Our violation-of-expectation (VOE) stimuli from Experiment 2 were selected from 2 large-scale procedurally generated video datasets, inspired by the infant cognition literature (Shu et al., 2021; Smith et al., 2019), and also contained 3 hand-animated scenarios from Experiment 1 (Fig. 1, panel A2). In total, there were 28^1^ scenarios. The 12 scenarios from the domain of physics featured inanimate objects, barriers, and rotating fans. In surprising events, solid objects passed through each other (*solidity*) or blipped in and out of existence (*permanence*) (Spelke et al., 1995). The 16 scenarios from the domain of psychology featured agents moving in physical environments, around physical obstacles, towards goal objects, and were further divided into scenarios involving surprising *actions* (12 scenarios), or surprising *environments* (4 scenarios) in which the actions occurred. In the psychological scenarios involving surprising actions, agents changed their goals (*goal*), or acted inefficiently (*efficiency*). In the psychological scenarios involving surprising environments, agents moved through an (apparently) solid wall (*agent-solidity*) (Saxe et al., 2006), or moved as though they were circumventing an obstacle, which was then missing (*infer-constraint*) (Csibra et al., 2003). Our primary analyses focus on the psychology-action events; in further exploratory analyses, we studied neural responses to the psychology-environment events. Expected and unexpected events within each domain were matched along an array of low-level visual features (Fig. S4). Independent adult observers rated the unexpected events from these three categories (physics, psychology-action, and psychology-environment) as equally surprising (Fig. S5).

Each VOE run had an event-related design (Fig. 1, panel B2): a 10 s rest period, 16 trials (6 physics, 6 psychology-action, and 4 psychology-environment), and then a final 10 s rest period, lasting a total period of approximately 7.0 minutes. All participants saw four runs, except for one participant in Experiment 2 who only underwent three runs due to time restrictions. Each trial had 4 parts: a familiarization movie (7.5 s), a corresponding test movie (7.5 s; either unexpected or expected), each followed by a 250 ms interstimulus interval, a fixation cross for a jittered duration of 4-10 s, and an attention check (2 s). Participants were asked to pay attention to the movies. During the attention check, they saw an image of an agent, object, or surface texture, and responded via button press to indicate whether that image appeared in the most recent trial. In anticipation that we may need to restrict our analysis to the first two runs, scenarios were split into two halves, one half assigned to runs 1-2 and the other assigned to runs 3-4, so that analyses over the first two runs would be conducted on the same stimuli across participants. We generated 128 unique random event sequences, one per run per participant, such that every run contained 8 unexpected and expected trials apiece, and the same number of physics (6), psychology-action (6), and psychology-environment (4) trials, and across sequences, each scenario appeared in each possible position within a trial an equal number of times. All participants underwent four runs.

### 2.4 Data acquisition

For full scanner protocols for both experiments, please see our pre-registration documents at https://osf.io/sa7jy/. In brief, for both experiments, neuroimaging data were acquired from a 3-Tesla Siemens Magnetom Prisma scanner located at the Athinoula A. Martinos Imaging Center of the McGovern Institute, using the standard 32-channel head coil. Participants viewed stimuli projected to a 12” x 16” screen behind the scanner, at a visual angle of approximately 14 x 19 degrees, through a mirror. Participants first underwent an anatomical scout scan (auto-align, acquired in 128 sagittal slices with 1.6 mm isotropic voxels, used to identify key anatomical landmarks and position the bounding box for subsequent anatomical and functional scans; TA = 0.14; TR = 3.15 ms; FOV = 260 mm), and a high-resolution MPRAGE anatomical scan (T1-weighted structural images acquired in 176 interleaved sagittal slices with 1.0 mm isotropic voxels, TA = 5:53, TR = 2530.0 ms; FOV = 256 mm, GRAPPA parallel imaging, acceleration factor of 2).

In Experiment 1, participants then underwent six runs of functional scans (gradient-echo EPI sequence sensitive to Blood Oxygenation Level Dependent (BOLD) contrast in 3 mm isotropic voxels in 46 interleaved near-axial slices covering the whole brain; EPI factor = 70, TR = 2 s, TE = 30.0 ms, flip angle = 90 degrees, FOV = 210 mm). Participants underwent four runs of the primary VOE task, followed by two runs of the DOTS localizer task. In total, the scanning session lasted about 60 minutes.

In Experiment 2, participants underwent 10 runs of functional scans (gradient-echo EPI sequence sensitive to Blood Oxygenation Level Dependent (BOLD) contrast in 3 mm isotropic voxels in 50 interleaved near-axial slices covering the whole brain; EPI factor = 70; TR = 2 s; TE = 30.0 ms; flip angle = 90 degrees; FOV = 210 mm). Six of these runs were dedicated to our 3 localizer tasks, two runs apiece. The remaining four runs were dedicated to our primary VOE task of interest. Participants underwent these tasks in a fixed order, with localizer runs interspersed between the VOE runs. In total, the scanning session lasted about 90 minutes.

### 2.5 Neuroimaging data pre-processing and analysis

Neuroimaging data were preprocessed using fMRIprep (Esteban et al., 2019) which is based on Nipype (Gorgolewski et al., 2011). Experiment 1 used fMRIprep version 1.2.6, and Experiment 2 used fMRIprep 2.0.2. In brief, the T1-weighted anatomical image was first skull-stripped; the resulting extracted image was segmented into cerebrospinal fluid (CSF), white matter (WM), and gray matter, and registered to the MNI152 template. Each series of functional data was skull-stripped, translational and rotational motion were estimated over the series, and slice-time correction was applied. Then, the resulting time series were resampled to native space, and then co-registered with the T1w reference image. Several confounding time-series were calculated, including framewise displacement (FD), the derivative of variance (DVARS) over frame-to-frame motion, and global signals within the CSF, WM, and whole-brain masks. Volumes were flagged as motion outliers if FD exceeded 0.5 mm or if DVARS exceeded 1.5. Physiological regressors were extracted using CompCor (Behzadi et al., 2007) Nuisance regressors were saved as outputs and passed to first-level modeling. Automatic removal of motion artifacts was conducted using independent component analysis, ICA-AROMA (Pruim et al., 2015) was performed on the preprocessed BOLD on MNI space time-series after removal of non-steady state volumes and spatial smoothing with an isotropic, Gaussian kernel of 6 mm FWHM (full-width half-maximum). For quality assurance, we visually inspected fMRIprep-produced report for each participant, and re-ran the pipeline when we noticed issues with the tissue segmentation or registration. See SM for full preprocessing pipeline details.

The preprocessed BOLD images for all tasks were analyzed using custom lab scripts using Nipype (Gorgolewski et al., 2011), which included run-level exclusion based on motion, first- and second-level modeling, and whole-brain analysis. Prior to first-level modeling, runs with more than 25% of frames exceeding motion of 0.4 mm based on framewise displacement were flagged and excluded from subsequent analyses. During run-level modeling, all regressors other than head movement parameters were convolved with a standard double-gamma hemodynamic response function, with a high-pass filter applied to both the data and the model. Event regressors were defined as a boxcar from the start and end of each block (localizer tasks) or event (VOE task). These first-level general linear models (GLMs) were then passed to subject-level and group-level analyses.

### 2.6 Localizing functional ROIs in individual participants

Our primary analyses relied on the subject-specific functional region of interest (ssfROI) approach (Fedorenko et al., 2010). The goal of this approach is to find, in individual participants, voxels that are maximally engaged during each of our hypothesized cognitive processes—social and physical prediction (identified using the DOTSloc task), early visual processing (identified using the motionLoc task), and goal-directed attention (identified using the spWMloc task)—while allowing the stereotactic location of the voxels selected to vary across people according to their unique neuroanatomy and functional organization.

#### 2.6.1 Regions of interest

Our analyses aimed to balance two considerations: to maximize sensitivity to responses in individual regions that are the best candidates for each of hypothesized mental processes, and to characterize the distribution of information across the cortex. Below, we first describe the procedures for constructing a large set of parcels for subsequent ROI analysis, and also procedures for selecting a small subset of parcels (“focal regions”) for the primary exploratory (Experiment 1) and confirmatory (Experiment 2) ROI analysis.

In total, we studied domain and event univariate effects in a set of 24 domain-general regions and 18 domain-specific regions. All of these regions were pre-registered ahead of data collection for Experiment 2. The full set of 24 domain-general regions included 20 multiple demand parcels constructed based on probabilistic maps from 197 adult participants engaging in the spWMloc task, by Fedorenko and colleagues (https://evlab.mit.edu/funcloc/). Four parcels for primary visual cortex and middle temporal area, which were anatomically defined based on the Desikan-Killiany and Destrieux cortical parcellations in Freesurfer (Pramod et al., 2022). (In Experiment 1, we did not have a localizer for MT, so we did not study this region.) Our domain-specific parcels were derived from group-level data from Experiment 1 on the DOTSloc task, runs 2-4 of the VOE task (reserving run 1 for studying the VOE response), and parcels for frontoparietal physics regions from Pramod et al. (2022), which respond more during judgments of the physical stability of block towers than judgments of the color of the blocks in the same stimuli. In brief, we intersected group maps from these data sources while avoiding overlap, and inflated the resulting parcels to create a more generous search space. In the end, we created 4 physical parcels that were derived from an intersection of the DOTSloc and frontoparietal parcels, 4 physical parcels that were derived from an intersection of the DOTSloc and VOE tasks (based on the physical > social contrast), and 10 social parcels that were derived from an intersection of the DOTSloc and VOE tasks (based on the social > physical contrast). For full details, see SM. These ROIs were pre-registered ahead of Experiment 2.

From this larger set of ROIs, we selected a few regions (“focal regions”) that served as the best proxies for each hypothesized cognitive process. For domain-specific psychological processing, we chose left and right superior temporal sulcus (STS). For domain-specific physical processing, we chose left and right supramarginal gyrus (SMG). Both regions were chosen based both on their domain-specific functions based on prior literature, and because in group-level analyses from Experiment 1, these regions showed greater responses to social and physical stimuli for both the VOE and the DOTSloc tasks (see SM Section 4). For domain-general visual processing, we chose bilateral primary visual cortex (V1) and bilateral middle temporal area (MT); because there was no independent localizer for area MT in Experiment 1, we studied left and right V1 as our two visual ROIs in Experiment 1. For domain-general goal-directed attention, we chose bilateral anterior parietal cortex (APC), and right precentral/inferior frontal cortex (RFC). These two MD regions, identified using runs 2-4 of the VOE task in Experiment 1, were chosen because they showed the biggest VOE effect size of all the MD ROIs, appeared in a meta-analysis over regions that encode reward prediction error during learning (Fouragnan et al., 2018), and are close in proximity to findings from previous research on neural responses to magic tricks and curiosity-inducing trivia (Kang et al., 2009; Parris et al., 2009)^2^. All bilateral parcels were made by combining the left and right hemisphere parcels for a given region. The RFC parcel was made by combining the two right precentral parcels from Fedorenko and colleagues. These ROIs were pre-registered as focal regions ahead of Experiment 2.

#### 2.6.2 ROI selection and extraction

See Figure 3B for a summary of the localizer tasks and contrasts used to identify ROIs. In both experiments, data used to select ROIs for individual participants were independent of the data used to evaluate their responses to the VOE stimuli. For both experiments, for each region, for each participant, we identified fROIs by using a localizer task to select the top 100 voxels within the corresponding parcel (i.e. those with the highest z values). See SM for more results showing the robustness of our findings to fROI size. In Experiment 1, fROIs for domain-specific regions were identified using the social versus physical interaction contrasts from the DOTSloc task, fROIs for MD regions were identified using the unexpected > expected contrast from runs 2-4 of the VOE task, and early visual ROIs were identified using the stimuli > rest contrast from the DOTSloc task. In Experiment 2, fROIs for domain-specific regions were identified using the social versus physical interaction contrast from the DOTSloc task, the MD fROIs were identified using the hard > easy contrast from the spWMloc task, MT was identified using the coherent > incoherent motion contrast in the motionLoc task, and V1 was identified using the stimuli > rest contrast from the spWM task.

### 2.7 Univariate analyses for psychology-action and physics events in focal regions

We first conducted (Experiment 1) and pre-registered (Experiment 2) a manipulation check to assess whether the neural VOE effect declined across experimental runs (see SM Section 6 for details). Following this procedure, we restricted our analyses to the first run of the VOE task in Experiment 1, and the first two runs of the VOE task in Experiment 2^3^.

Throughout the methods and results, we will refer to neural VOE effects (i.e., unexpected vs. expected) as “event” effects, and neural domain effects (i.e., psychology vs. physics) as “domain effects.”

For each ROI, we modeled the average response per region as predicted by a main effect of domain, a main effect of event, and the interaction across them, including a random effect to account for correlated data within individual people: meanbeta ~ domain * event + (1|subjectID). We used the lme4 and lmerTest packages (Bates et al., 2015; Kuznetsova et al., 2017) to conduct this analysis, and the lsmeans package (Lenth, 2016) to extract pairwise comparisons of response magnitudes for each domain. We calculated a Bayes Factor (BF) for each effect based on the Bayesian Information Criterion (BIC) from two models: one model containing that predictor (*BIC_0*), and a second model without that predictor (*BIC_1*) (*BF=e^(BIC_0 - BIC_1) / 2*; Wagenmakers, 2007). For example, to calculate the BF for a domain main effect, we took BICs from a model including that main effect (*meanbeta ~ domain + event + (1|subjectID)*) and the same model excluding that main effect (*meanbeta ~ event + (1|subjectID)*). To compute BFs for the interaction between domain and event, we compared a model with the interaction (meanbeta ~ domain * event + (1|subjectID)) to a model that contained the same predictors as main effects (*meanbeta ~ domain + event + (1|subjectID)*). Details about implementation can be found in our openly available analysis scripts, at https://osf.io/sa7jy/.

First, we studied the responses of each ROI to the primary VOE task 8 focal regions. For both experiments, we focused on the unexpected and expected test events from physical scenarios and psychological scenarios involving surprising actions (physics and psychology-action events; Fig. 1A-B). Our significance threshold for analyses in focal regions was α = .025, two-tailed (correcting for 2 regions per ROI type).

Then, we tested in an exploratory analysis whether any of our results are explained by variability in the lower-level visual statistics in our stimuli (e.g., motion, spatial extent). We focused this analysis on Experiment 2 which had many more unique stimuli than Experiment 1 and therefore could support the full set of visual features as predictors. Full details about this analysis can be found in the SM. In brief, for each video, we calculated the amount of luminance, contrast, motion, high spatial frequency, low spatial frequency, curvilinearity, and rectilinearity. We then z-scored the scores for each feature across videos. Lastly, we refit the mixed effects models for each region including these features as additional regressors, and evaluated whether the (positive and negative) effects we observed in our focal regions held, after accounting for variance in low-level stimulus features.

### 2.7 Univariate analyses for psychology-action and physics events in broader set of ROIs

In addition to our focal regions, we also wanted to characterize the responses of regions across the cortex. Thus, as a complementary approach, in further exploratory analyses, we studied domain and event univariate effects in our larger set of 18 domain-specific regions and 24 domain-general regions. The voxel selection procedure was identical to the confirmatory analyses, except that focal ROIs that were combined (e.g., bilateral V1, which contains voxels from both left and right V1; and RFC, which contains voxels from two precentral multiple demand masks from Fedorenko et al.) were split to maximize the number of regions for subsequent analyses. All of these ROIs were pre-registered ahead of Experiment 2. We studied the responses in these regions in two ways.

First, we looked in each region for evidence of a domain effect, event effect, and interactive effect, with a conservative significance threshold to account for the number of regions we explored (Bonferroni correction; ɑ = .002 for 24 domain-general regions; ɑ = .003 for 18 domain-specific regions). Second, we conducted whole-brain group random-effects analyses, which we report in the SM.

Second, we conducted an analysis investigating the organization of event and domain information across this broader set of domain-specific and domain-general regions^4^. In this analysis, we ask: For a set of regions, can we predict the size of a region’s domain preference during expected events from that region’s domain preference during unexpected events? Can we predict the size of a region’s event preference for physical events from that region’s event preference for psychology-action events? And do the answers to these questions differ depending on whether the regions in question are domain-specific or domain-general? One possibility is that domain-general regions are organized by event (i.e., show predictable preferences for unexpected vs. expected events, across domains, across regions), and domain-specific regions are organized by domain (i.e., show predictable preferences for psychology-action vs. physics events, across unexpectedness, across regions). Another possibility is that the responses in these regions go beyond the information they were defined over: for example, domain-general regions were defined as those maximally engaged for attentional demand and visual processing, but could still show systematic domain preferences; domain-specific regions were defined as those maximally engaged during social versus physical prediction, but could still show systematic VOE effects across domains.

We conducted this analysis separately for domain-specific and domain-general regions. The steps of this analysis were as follows: First, we computed four effect sizes (standardized betas) per region: the magnitude of the domain preference for expected events (*β_domain_expected*) and for unexpected events (*β_domain_unexpected*), and the magnitude of the event preference for psychology-action events (*β_event_psychology*) and separately for physics events (*β_event_physics*). For Experiment 2, these effects were extracted from statistical models that controlled for low-level visual features. Given these four effect sizes per region, we next asked whether each group of regions showed systematic responses to domains (i.e., preferences for psychology-action or physics events, across event types), and systematic responses to events (i.e., preferences for unexpected over expected events, across domains). For each set of regions, we computed two correlation values. The first correlation expresses the correspondence between preferences for domains across events types, across regions (*r_domain=cor(β_domain_expected, β_domain_unexpected)*), and the second correlation expresses the correspondence between preferences for events across domains, across regions (*r_event=cor(β_event_psychology, β_event_physics)*). Correlations were calculated using nonparametric tests of independence, which test the null hypothesis that two vectors are statistically independent, but do not assume the linearity of their dependence (α = .05, two-tailed). Our prediction for Experiment 2 was that both domain-specific and domain-general regions would show systematic preferences for domains (*r_domain*), more so than by event (*r_event*). To test the hypothesis *r_domain* will be larger than *r_event,* we computed the bootstrapped difference between these two values under the null hypothesis (4000 iterations). The p-value was the proportion of bootstrapped observations that were equal to or exceeded the empirical difference between *r_domain* and *r_event* (α = .05, one-tailed).

### 2.8 Multivariate analyses for psychology-action and physics events

In addition to these univariate analyses, we pre-registered and conducted a series of multivariate pattern analyses (MVPA). We tested whether any of our focal regions contained distinct patterns of activity for unexpected versus expected events (and if so, whether these patterns were domain-specific or domain-general). For both Experiments 1 and 2, we used the data from the same ROIs as the univariate analysis, but instead of averaging responses across voxels, we calculated the Euclidean distance for each participant for each ROI along the following category boundaries: events across domains, domains across events, events within domains (i.e., information about event within psychology-action and physics separately), and domains within events (i.e., information about domains within unexpected and expected events separately). To evaluate whether a given region had multivariate information about a given category boundary, we first computed the within- versus between-category distance for each boundary. Then, we tested whether the within-category distances were significantly less than the between-category distances using a one-tailed Wilcoxon signed rank test (α = .025, one-tailed, correcting for 2 regions per ROI type).

### 2.9 Univariate analyses for psychology-environment events

In further exploratory analyses for Experiment 2, we studied the responses of these regions to surprising physical outcomes revealed by an agent’s actions (psychology-environment; Fig. 6). To do this, we fit a linear mixed-effects model on neural responses predicted by event type, with a random intercept for subject (*meanbeta ~ event + (1|subjectID)*). We conducted this analysis both over the first two runs of the VOE task (to match the results for the psychology-action and physics events), and over all four runs (because we did not observe habituation of the VOE effect in these events; see SM Section 6 and Fig. S2).

## 3 Results

### 3.1 Results in focal regions

**Fig. 4.**
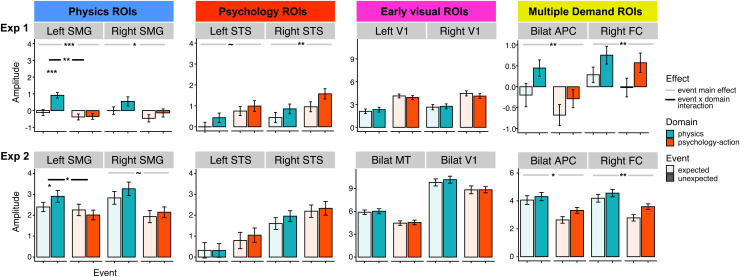
Results of univariate subject-specific functional regions of interest (ssfROI) analysis from Experiment 1 (exploratory) and Experiment 2 (confirmatory) in domain-specific regions (first two columns: left and right superior temporal sulcus, STS, and left and right supramarginal gyrus, SMG), domain-general early visual regions (bilateral primary visual cortices, V1, and bilateral motion-sensitive area, MT), and domain-general multiple demand regions (bilateral anterior parietal cortices, APC, and right frontal cortex, RFC). Y axis indicates the average beta (i.e., amplitude of response) per region, relative to fixation/rest, across 17 participants (Experiment 1) and 32 participants (Experiment 2). Error bars indicate the standard error of the mean, taking into account within-subjects variance. P values (~p < 0.10, *p < 0.05, **p < 0.01, ***p < 0.001, two-tailed) come from mixed effects models including main effects for event and domain, as well as their interaction. For simplicity we only show main effects for event (unexpected versus expected) and the interaction between event and domain; see main text for results regarding main effects of domain. See Figure 3 and main text for information about how these regions were functionally localized in individual participants.

In a first set of analyses, we studied neural responses in a small number of regions that served as proxies for each of our hypothesized cognitive processes (psychological and physical prediction, early visual processing, endogenous attention). See Figure 4 full regression tables for all analyses from all regions are available in the SM.

First, we tested the hypothesis that the VOE response is supported in part by domain-specific processing. Do we find evidence for domain-specific processing of violations of physical and psychological expectations, in cortical regions selective for those domains?

#### 3.1.1 Physics ROIs

In Experiment 1, we first confirmed the selectivity of left and right SMG for physical over social stimuli: Both left and right SMG responded preferentially to physical events (left SMG: 95% CI = [0.252, 0.432], unstandardized B coefficient = 0.342, p-value < 0.001, two-tailed, Bayes Factor (BF) > 1000; right SMG: [0.169, 0.365], B = 0.267, p < 0.001, two-tailed, BF > 1000). Then, we conducted the key test for physical prediction error. We found that left SMG showed a VOE response that differed across domains (domain x event interaction effect: [0.104, 0.397], B = 0.25, p = 0.001, two-tailed, BF = 2.758). LSMG responded more to unexpected than expected physical events (B = 1.031, p = <0.001, two-tailed), but did not distinguish between unexpected and expected psychological events (B = 0.03, p = 0.888, two-tailed). RSMG showed a main effect of event ([0.039, 0.399], B = 0.219, p = 0.018, two-tailed, BF = 0.231), but no significant interaction between event and domain ([-0.123, 0.237], B = 0.057, p = 0.535, two-tailed, BF = 0.017).

We then pre-registered the prediction for domain-specific prediction error in left SMG in Experiment 2. Like in Experiment 1, both left and right SMG responded more to physical than psychological events (left SMG: [0.104, 0.412], B = 0.258, p = 0.001, two-tailed, BF = 2.185; right SMG: [0.336, 0.672], B = 0.504, p < 0.001, two-tailed, BF > 1000). We again found that left SMG showed a signature of domain-specific prediction error: an interaction between event and domain ([0.104, 0.412], B = 0.258, p = 0.001, two-tailed, BF = 2.185), with greater responses for unexpected than expected physical events (B = 0.51, p = 0.023, two-tailed), and no significant VOE effect for psychological events (B = -0.241, p = 0.28, two-tailed). Right SMG showed a marginally higher response to unexpected events regardless of domain ([-0.007, 0.329], B = 0.161, p = 0.062, two-tailed, BF = 0.078), with no interaction between event and domain ([-0.111, 0.224], B = 0.057, p = 0.511, two-tailed, BF = 0.017).

#### 3.1.2 Psychology ROIs

In Experiment 1, we found that both left and right STS responded more to psychological than physical events (left STS: [-0.509, -0.148], B = -0.329, p < 0.001, two-tailed, BF = 6.951; right STS: [-0.699, -0.317], B = -0.508, p < 0.001, two-tailed, BF > 1000). However, we did not find evidence for a distinctively psychological prediction error—an interaction between event and domain—in these regions (left STS: [-0.131, 0.23], B = 0.05, p = 0.593, two-tailed, BF = 0.016; right STS: [-0.218, 0.116], B = -0.051, p = 0.553, two-tailed, BF = 0.016) Instead, we found that the right STS responded more to unexpected events from both domains ([0.091, 0.425], B = 0.258, p = 0.003, two-tailed, BF = 1.177). The left STS showed a similar, though marginal, main effect of event ([-0.012, 0.349], B = 0.168, p = 0.07, two-tailed, BF = 0.074).

In planning for Experiment 2, in which we plausibly had greater statistical power (due to the larger sample size, more stimuli, and more runs of data), we pre-registered two alternative hypotheses: that the STS would show domain-specific psychological prediction error, which would lead to an interaction between event and domain, or that the STS encodes both physical and psychological information relevant for action understanding, which would lead to a main effect of domain, and of event, but no interaction effect. In the confirmatory analyses of Experiment 2, we found support for neither hypothesis. Both left and right STS responded more to psychological events (left STS: [-0.491, -0.109], B = -0.3, p = 0.002, two-tailed, BF = 1.584; right STS: [-0.405, -0.08], B = -0.242, p = 0.004, two-tailed, BF = 0.861). However, neither left nor right STS responded more to unexpected than expected events (left STS: [-0.129, 0.253], B = 0.062, p = 0.524, two-tailed, BF = 0.019; right STS: [-0.039, 0.286], B = 0.123, p = 0.139, two-tailed, BF = 0.039), and there was no interaction between domain and event in these regions (left STS: [-0.257, 0.125], B = -0.066, p = 0.501, two-tailed, BF = 0.019; right STS: [-0.109, 0.216], B = 0.054, p = 0.517, two-tailed, BF = 0.016). Results were similar when we defined STS ROIs not based on the external localizer, but rather, as voxels that responded more to psychological than physical VOE events (see SM for details). Thus, we did not find consistent evidence for domain-general or domain-specific psychological prediction error in our focal psychology ROIs.

Next, we tested for evidence for domain-general processing of violations of expectation, in cortical regions associated with visual processing and endogenous attention.

#### 3.1.3 Early visual ROIs

In Experiment 1, we found via exploratory analyses that neither left nor right V1 responded more to unexpected than expected events (left V1: [-0.222, 0.23], B = 0.004, p = 0.973, two-tailed, BF = 0.018; right V1: [-0.293, 0.174], B = -0.06, p = 0.618, two-tailed, BF = 0.021). Both left and right V1 responded more to psychological events (left V1: [-1.13, -0.678], B = -0.904, p < 0.001, two-tailed, BF > 1000; right V1 [-1.022, -0.555], B = -0.788, p < 0.001, two-tailed, BF > 1000).

In Experiment 2, we found that neither bilateral V1 nor bilateral MT responded differentially to unexpected and expected events (V1: [-0.171, 0.356], B = 0.093, p = 0.492, two-tailed, BF = 0.027; MT: [-0.079, 0.187], B = 0.054, p = 0.428, two-tailed, BF = 0.015). Both bilateral V1 and bilateral MT responded more to physical than psychological events (the opposite effect from that in Experiment 1) (V1: [0.312, 0.839], B = 0.575, p < 0.001, two-tailed, BF = 145.691; MT: [0.594, 0.86], B = 0.727, p < .001, two-tailed, BF > 1000). The higher average response to physical events in MT appears to be driven by variance in low-level statistics in the stimuli (see Section 3.2 and SM for details); after controlling for these features, MT no longer showed a significant domain preference ([-0.01, 0.389], B = 0.19, p = 0.064, two-tailed). V1 continued to show a preference for physical events, after accounting for these same features ([0.122, 0.813], B = 0.468, p = 0.008, two-tailed)

We conducted an additional exploratory analysis, reported in the SM, confirming that these early visual regions *do* respond to visually novel events (familiarization events, relative to test events). Thus, early visual regions are sensitive to stimulus-driven novelty, but do not respond more to unexpected events, and do not respond consistently to stimuli from either domain.

#### 3.1.4 Goal-directed attention ROIs

Lastly, we tested the hypothesis that the VOE response is (also) supported by domain-general endogenous attention by studying responses in two multiple demand regions: the right frontal cortex (RFC) and bilateral anterior parietal cortex (APC; see SM for evidence for low overlap with SMG ROIs in individual participants).

In Experiment 1, we found via exploratory analyses that right frontal cortex (RFC) responded more to unexpected than expected events (main effect of event: [0.097, 0.434], B = 0.265, p = 0.002, two-tailed, BF = 1.381). This region did not respond preferentially to physical or psychological events (main effect of domain: [-0.047, 0.291], B = 0.122, p = 0.16, two-tailed, BF = 0.036), and there was no interaction between event and domain ([-0.2, 0.138], B = -0.031, p = 0.719, two-tailed, BF = 0.014). We found that bilateral anterior parietal cortex (APC) also responded more to unexpected than expected events (main effect of event: [0.096, 0.422], B = 0.259, p = 0.002, two-tailed, BF = 1.453), and responded more to physical than psychological events (main effect of domain: [0.14, 0.466], B = 0.303, p < 0.001, two-tailed, BF = 7.935), with no interaction between domain and event ([-0.099, 0.227], B = 0.064, p = 0.447, two-tailed, BF = 0.017).

We then pre-registered these same predictions in Experiment 2. We found that both RFC and APC responded more to unexpected than expected events (RFC: [0.103, 0.48], B = 0.291, p = 0.003, two-tailed, BF = 1.36; APC: [0.032, 0.436], B = 0.234, p = 0.024, two-tailed, BF = 0.208), with no intersection between domain and event (RFC: [-0.297, 0.08], B = -0.109, p = 0.261, two-tailed, BF = 0.028; APC: [-0.312, 0.092], B = -0.11, p = 0.287, two-tailed, BF = 0.029). Both RFC and APC responded more to physical than psychological events (RFC: [0.407, 0.783], B = 0.595, p < 0.001, two-tailed, BF > 1000; APC: [0.401, 0.804], B = 0.602, p < 0.001, two-tailed, BF > 1000). The higher average response to physical events in both regions appears to be driven by variance in low-level statistics in the stimuli (see Section 3.2 and SM for details); after controlling for these features, neither region showed a domain preference (RFC: [-0.006, 0.577], B = 0.286, p = 0.056, two-tailed; APC: [-0.156, 0.426], B = 0.135, p = 0.365, two-tailed).

To summarize, MD ROIs did not respond differentially to physical and psychological events, but did respond more to unexpected events across domains.

### 3.2 Controlling for visual statistics

We tested in an exploratory analysis whether any results (domain-specific event response in LSMG, domain-general event responses in RFC and APC) from Experiment 2 are explained by variability in the lower-level visual statistics in our stimuli (e.g., motion, spatial extent). We found that after accounting for variability in the contrast, luminance, motion, spatial frequency content, rectilinearity, and curvilinearity of the stimuli, all positive and negative VOE effects from our confirmatory (Experiment 2) analyses held, including the null findings in V1, MT, LSTS, and RSTS, as well as the positive effects in LSMG, RFC, and APC. The domain preferences in three domain-specific regions (RSMG, LSTS, RSTS) also remained significant, after controlling for the visual features. The domain preferences for V1 held after controlling for these features, but the direction of these preferences was inconsistent across experiments and stimuli. The apparent preferences for physical events in the two MD regions (RFC and APC) and one visual region (MT) were no longer significant, after controlling for visual features. See SM for details.

### 3.3 Exploring domain and event effects in additional ROIs

As a complementary approach to our analyses in a small set of focal regions, in further exploratory analyses, we studied domain and event univariate effects in a larger set of 18 domain-specific regions and 24 domain-general regions (22 in Experiment 1; minus left and right MT). None of the additional regions we explored, in either experiment, showed a significant VOE effect, though many showed differential responses to physical and psychological events. See SM for results from these analyses, as well as results from whole-brain analyses.

Finally, we conducted a series of analyses investigating the reliability of event and domain information across domain-specific and domain-general regions. Are domain-specific regions and domain-general regions organized by domain and event, respectively? Or do the responses in these regions go beyond the information they were defined over (attentional demand and visual processing for domain-general regions; social vs. physical prediction for domain-specific regions)?

**Fig. 5.**
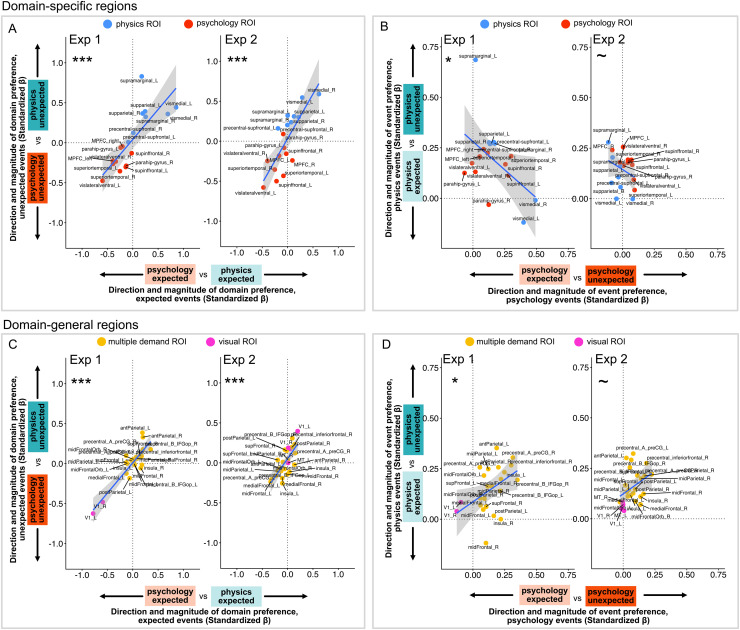
Organization of domain and event information in domain-specific regions (A-B) and domain-general regions (C-D). Each dot indicates the size of a single region’s preference for psychological and physical domains (A, C), or for unexpected versus expected events (B, D), in standardized betas. In Panels A and C, a value of 0 indicates equal responses to expected psychological and expected physical events (x-axis), or to unexpected psychological and unexpected physical events (y-axis); in panels B and D, a value of 0 indicates equal responses to expected psychological and unexpected psychological events (x-axis), or to expected physical and unexpected physical events (y-axis). Each panel shows the correspondence between the size of two effects across regions: either the correspondence between preferences for psychological and physical domains across unexpected and expected events (A, C), or between preferences for unexpected and expected events across the psychological and physical domains (B, D). Effect sizes from Experiment 2 were derived from statistical models that controlled for low-level visual statistics. Line of best fit is for illustrative purposes; significance indicators (~p < 0.10, *p < 0.05, ***p < 0.001, two-tailed) come from non-parametric test for independence for each panel. All of these regions were pre-registered ahead of Experiment 2.

Across both Experiments 1 and 2, we found that response magnitude across 24 putatively domain-specific regions is reliable by domain, but not by event. For these regions, the size of a region’s domain effect (psychology vs. physics) for expected events strongly predicts the size of the same region’s domain effect for unexpected events (Experiment 1: r = 0.791, p < 0.001; Experiment 2: r = 0.799, p < 0.001). However, the size of a region’s VOE effect (unexpected vs. expected) for psychology-action events was weakly anticorrelated with the size of the same region’s VOE effect for physics events (Experiment 1: r = -0.476, p = 0.044; Experiment 2: r = -0.316, p = 0.098). The reliability of domain information was greater than for event information (bootstrapped difference in correlations; Experiment 1: 95% CI [0.7, 1.556], p = 0.001; Experiment 2: 95% CI [0.568, 1.511], p < 0.001). See Figure 5A-B. Thus, this set of domain-specific regions show systematic and predictable responses to psychological and physical events that generalizes across expected and unexpected events.

What about domain-general regions, that were defined based on responses to visual information (V1 and MT) or to a spatial working memory task (MD regions), with no reference to domain information? We found that responses from these regions were reliable for both domain and event contrasts, in both Experiments 1 and 2. Across these regions, the domain effect (psychology vs. physics) for expected events strongly predicted the domain effect for unexpected events (Experiment 1: r = 0.876, p < 0.001; Experiment 2: r = 0.736, p < 0.001). In addition, the psychology event effect (unexpected vs. expected) positively predicted the physical event effect (Experiment 1: r = 0.449, p = 0.034; Experiment 2: r = 0.367, p = 0.037). Like in domain-specific regions, the reliability of domain information was greater than the reliability of event information (bootstrapped difference in correlations; Experiment 1: (95% CI [0.105, 0.617], p = 0.003); Experiment 2: (95% CI [0.122, 0.789], p = 0.005). See Figure 5C-D. Thus, this set of domain-general regions shows systematic and predictable responses to psychological and physical events, that generalizes across expected and unexpected events, and also show predictable responses to unexpected and expected events, that generalizes across domains. Although domain information was not used to define these ROIs, and although these regions are typically thought to be domain-general, this result suggests that domain information still organizes the pattern of univariate responses in this set of regions.

### 3.4 Multivariate tests of event and domain information

In addition to these univariate analyses, we pre-registered and conducted a series of multivariate pattern analyses (MVPA). We tested whether any of our focal regions contained distinct patterns of activity for unexpected versus expected events (and if so, whether these patterns were domain-specific or domain-general). By contrast to the univariate results, we found no evidence for a consistent spatial pattern distinguishing unexpected versus expected events in any region, and at the same time, strong evidence for consistent spatial patterns distinguishing between domains in many of our focal regions. This null result held even though we used Euclidean distance as the distance metric, which takes response magnitude into account. The dissociation between univariate and multivariate information was unexpected to us; we will speculate about possible implications of this result in the General Discussion. The full multivariate results are presented in the SM.

### 3.5 Neural responses to physically surprising outcomes, revealed by an agent’s action

**Fig. 6.**
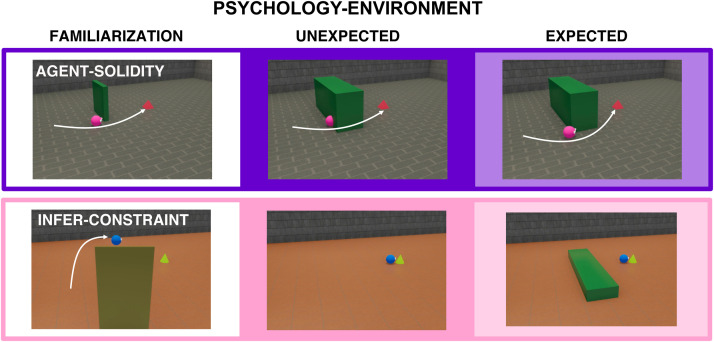
Stimuli from the domain of intuitive psychology, wherein the actions of an agent revealed a surprising physical outcome in the surrounding environment (psychology-environment). In *agent-solidity*, an agent passes through a solid wall; in *infer-constraint*, an obstacle that explains an agent’s action is missing.

In our primary analyses, reported above, we studied neural responses to surprising actions (psychology-action events). How do our focal domain-specific and domain-general regions respond to surprising events involving both agents and objects (psychology-environment scenarios, Fig. 6), wherein a physical outcome is rendered surprising in light of an agent’s action? To ask this question, in exploratory analyses we modeled the responses of all the focal regions towards the 4 psychology-environment scenarios from Experiment 2. When restricting the analysis to data from the first two runs, like in our confirmatory analyses in psychology-action and physics events, the only focal ROI that showed a VOE effect was the right STS ([0.095, 0.542], B = 0.319, p = 0.006, two-tailed, BF = 1.156).

Further exploratory analyses over all runs of the experiment suggested that many focal ROIs showed strong VOE effects towards these stimuli across runs, including domain-specific physics ROIs (left SMG: [0.229, 0.469], B = 0.349, p < 0.001, two-tailed, BF > 1000; right SMG: [0.156, 0.415], B = 0.285, p < 0.001, two-tailed, BF = 65.936), a domain-specific psychology ROI (right STS: [0.159, 0.342], B = 0.25, p < 0.001, two-tailed, BF > 1000) and MD ROIs (APC: [0.034, 0.293], B = 0.163, p = 0.014, two-tailed, BF = 0.146; RFC: [0.079, 0.34], B = 0.209, p = 0.002, two-tailed, BF = 0.934), though not early visual regions (bilateral V1: [-0.064, 0.203], B = 0.07, p = 0.306, two-tailed, BF = 0.012; bilateral MT: [-0.009, 0.175], B = 0.083, p = 0.078, two-tailed, BF = 0.023), nor left STS ([-0.084, 0.209], B = 0.063, p = 0.404, two-tailed, BF = 0.011). These effects were spatially consistent across participants, appearing in the SMG and STS in whole-brain random effects analyses. See Figure S11.

## 4 Discussion

Why do infants look and attend to surprising events, like when a ball (apparently) passes through a solid wall? The underlying mental processes that guide looking to events like these remain controversial, despite decades of behavioral studies. Thus in the current work, we used the tools of cognitive neuroscience to directly and simultaneously examine these mental processes, albeit in adults. We localized the brain regions in individual adult participants that support domain-specific and domain-general processes hypothesized to account for VOE (domain-specific psychological and physical reasoning, domain-general visual prediction error, and domain-general task-driven attention), and tested which of these processes show a corresponding neural VOE effect for stimuli from classic infant experiments. Overall, we found evidence that unexpected events in these stimuli (i) did not evoke processes similar to early-stage visual processing, (ii) evoked processes similar to endogenous goal-driven attention, and (iii) for physical events, evoked distinctively physical processing, in adult brains.

Before we discuss our positive findings, let us consider the implications of our negative findings from early visual regions. We found no evidence for the hypothesis that VOE stimuli evoke responses associated with visual processing of novel visual features. Primary visual cortex (V1) and motion-sensitive area (MT), did not respond more to unexpected than expected VOE events: The voxels that, in individual participants, were maximally responsive to visual stimuli (in V1) or to coherent motion (in MT), responded equally to unexpected and expected scenarios, regardless of domain. This result provides evidence against the hypothesis that unexpected events in infant studies attract attention merely because they contain an array of novel low-level visual features (Bogartz et al., 2000; Rivera et al., 1999), because V1 and MT should be sensitive to exactly these features.

Next, we consider the implications of the findings from domain-specific physical and psychological regions, and domain-general multiple demand regions, for VOE in human adults.

### 4.1 Violations of physical expectations

What happens in the minds and brains of adults when they see a violation of object support, solidity, or permanence? Prior research proposes that people possess a system for “intuitive physics” (Battaglia et al., 2013; Ullman et al., 2017): a capacity to represent the visual world in terms of the objects and surfaces in it, including inductive biases that objects are permanent and solid, that allows adults to form expectations about what will happen next, and to detect deviations from those expectations. Prior work suggests both a distinctive neural source of these capacities (Fischer et al., 2016; Pramod et al., 2022; Schwettmann et al., 2019), as well as early emergence in behavioral studies of infants (Baillargeon, 1995). Our results suggest that when adults see a physically surprising event, it evokes both a prediction error within that system for intuitive physics (supported by the frontoparietal physics network, including the SMG), and also a domain-general orienting response towards that event (supported by multiple demand regions, including the APC and RFC).

Whereas in behavioral research, domain-general and domain-specific contributions to the VOE effect are difficult to separate, neuroimaging allowed us to identify both domain-specific and domain-general correlates of the VOE effect simultaneously. Having found evidence for both processes, many questions follow. One question is whether physical prediction error is initially computed in one region, and passed to the other(s), and if so in which direction. Does a physical prediction error signal arise initially in LSMG, which is then read out by RFC or APC? Or does the LSMG pass physically relevant information to MD regions, and then receive a signal of physical prediction error from these regions? These questions are best addressed using neuroimaging techniques with good temporal resolution, like electroencephalography (EEG) and magnetoencephalography (MEG).

Another question is whether the neural population code for unexpectedness in MD regions, like RFC and APC, is truly domain-general. When we measured the reliability of domain and event univariate responses in domain-general regions, we found that across 24 regions, the strength of an MD region’s VOE effect generalized (albeit weakly) across domains. However, we could not test whether the pattern of response to physically unexpected events could be used to decode psychologically surprising events, or vice versa, because we could not measure reliable spatial patterns distinguishing expected vs unexpected events. By contrast, there were consistent patterns of information distinguishing the physical and psychological events, both within and across event types, in many regions (see SM for details). In sum, unexpected events led to greater activity in MD regions, but not in a consistent spatially structured manner. In this way, our results are consistent with prior evidence that prediction error increases response magnitude but reduces population code precision (Kok et al., 2016; Koster-Hale & Saxe, 2013). If this interpretation is true, then MVPA cannot be used to test hypotheses about the representations underlying VOE responses, at least the way they are conceptualized in the current research.

What can we infer or predict about infant brains, given these findings from adults? Studying the brains of adults to evaluate hypotheses about neural function and behavior in infants has both strengths and limitations. One strength is that studying adults allowed us to be more confident about the functions of the regions we studied, by using validated localizer tasks that targeted each candidate mental process underlying VOE. This design was possible because adults can tolerate long scans and can be instructed to perform tasks in the scanner. It is much harder to design and run localizer tasks in infants, but without localizers, reverse inference over functional activation alone is not straightforward (Fedorenko, 2021; Poldrack, 2006) (e.g., in the APC and SMG, which occupy approximately the same cortical territory across people, but are spatially and functionally distinct in individual adults; see SM). In our experimental design, we prioritized stimuli and procedures that correspond to prior studies of infants. One weakness of this strategy was that these may have not been the ideal conditions for maximizing adult engagement: adults’ neural VOE effect quickly habituated over just a few experimental runs (see SM for details). A second weakness is that showing adults infant-friendly stimuli does not guarantee that adults and infants would attend to the same portions of these stimuli. To us, the simple nature of these events (just one or two objects moving; just one agent acting) makes it more likely that both adults and infants would display similar patterns of looking, though this prediction merits further study.

Prior neuroimaging studies suggest that infants have similar organization of large-scale cortical networks, as well as similar cortical responses evoked by agents and objects, to those of adults (Dehaene-Lambertz & Spelke, 2015; Eyre et al., 2021; Grossmann, 2015; Hyde et al., 2018; Kosakowski, Cohen, Takahashi, et al., 2021; Lloyd-Fox et al., 2009; Powell et al., 2017). Thus, we speculate that all the focal regions we studied in adults are present in approximately the same locations, and functional, in infants under one year of age. Most relevantly, work using near-infrared spectroscopy in 5- to 7-month-old infants reported increases in activity in parietal cortex when infants saw objects move in a discontinuous path, or change speed (Wilcox et al., 2010). If multiple demand and frontoparietal physics regions could be separated and studied in infant brains, then we predict that violations of physical expectations would evoke activity in domain-specific and domain-general regions in infants, just as in adults, and that both would contribute to infant looking behavior in VOE studies.

### 4.2 Violations of psychological expectations

How do the human adult mind and brain respond to deviations from efficient or goal-directed action? In addition to capacities for physical understanding, prior research shows that people have an intuitive theory of rational action (Baker et al., 2009; Jara-Ettinger et al., 2016): a capacity to represent people as agents with mental states who plan intentional actions at a cost to themselves, which allows adults to predict and explain other agents’ behaviors. These capacities emerge in infancy (Baillargeon et al., 2016; Gergely & Csibra, 2003; Liu et al., 2017), and are likely supported by cortical regions involved in action processing and social cognition (Saxe, Carey, et al., 2004).

In the current study, apparently irrational actions evoked increased activity in regions engaged by endogenous attention, suggesting that psychological prediction error like physical prediction error, leads to a domain-general orienting response. However, the existence of domain-specific psychological prediction error, and the role of the STS were less clear. In our study, STS responded to the actions of agents, consistent with the social functions of the superior temporal sulcus (Deen et al., 2015; Gao et al., 2012; Isik et al., 2017; Saxe, Xiao, et al., 2004; Shultz et al., 2011; Vander Wyk et al., 2009). However, evidence of social prediction error in the STS was less conclusive. Prior literature is similarly mixed, with some researchers finding activation in the STS for violations of rational action (Brass et al., 2007; Jastorff et al., 2011; Marsh et al., 2014; Shultz et al., 2011; Vander Wyk et al., 2009), and others finding activation in frontoparietal regions that could reflect the same responses we measured in APC and RFC (Marsh et al., 2014; Ramsey & Hamilton, 2010; Southgate et al., 2014). Thus, while the STS is likely involved in the processing of social information more broadly, it is unclear how the STS is involved in expressing an intuitive theory of action, including prediction error over that theory. One possibility is that the STS does encode prediction errors over action, but shows a more sustained response for action outcomes that are harder to explain away (e.g., from prior work, when a person opens a door with her knee, even though her hands are free, Brass et al., 2007; a person expressing disgust at an object, and then reaching for it, Vander Wyk et al., 2009. By contrast, the actions we tested here and in prior work (Ramsey & Hamilton, 2010) were much simpler (someone changing their mind about which object to pursue; someone taking an indirect direct path to goal) and easier to explain away, which could have led to a smaller STS VOE response. Another possibility is that another region, for example in the action observation network (Caspers et al., 2010), would encode action-specific prediction error; however, our exploratory results in some of these regions found no evidence for this prediction (see SM).

As early as can be measured, activity in the superior temporal cortex is evoked by social stimuli in infants as well as adults, responding to faces (Kosakowski, Cohen, Herrara, et al., 2021; Lloyd-Fox et al., 2009), actions (Biondi et al., 2016), and social interactions (Biondi et al., 2021; Hakuno et al., 2020; Lloyd-Fox et al., 2015). But, like in adults, the STS in infants may not encode action prediction errors during simple scenes involving violations of rational action. In one near-infrared spectroscopy (NIRS) study with 9-month-old infants, Southgate et al. (2014) measured responses from the temporal and parietal cortex while infants watched an animated agent move towards one object, and then move towards the same object in a new location or move towards the same location, now occupied by a new object, much like our goals task. The authors found that two contiguous channels over the left anterior parietal cortex responded to changes in an agent’s goals (which could correspond to the responses we measured in adult APC); no other contiguous channels showed a similar response. Based on these observations, we predict that infant looking to VOE events involving surprising actions will reflect both domain-specific and domain-general neural sources, though it is an open question whether the STS in infants encodes action prediction errors.

### 4.3 Distinct and shared representations across intuitive physics and psychology

Our study found evidence supporting the broad division between the physical and psychological domains in the human brain. First, many of the 42 regions we studied preferentially responded to events involving agents or objects; this was true both for domain-specific regions we defined based on a social vs physical contrast, and also for domain-general regions we defined based on a hard versus easy attentional demand contrast (Fig. 5A, 5C). Furthermore, we found that in domain-specific regions, VOE effect sizes tended to trade off between domains: Regions that tended to show a VOE effect in one domain tended not to show that effect in the other domain.

However, in some ways, our results also highlight the interactions between these two domains. First, physical outcomes that were surprising in light of observed actions (Fig. 6) evoked activity in both psychological and physical ROIs. These events plausibly required computations from both domains. It is not surprising, by itself, to see an agent move on a straight path, but it is surprising if that path is through a solid object. It is not surprising, by itself, to see an occluder reveal empty space, but it is surprising if an obstacle, implied by an agent’s action, is not there. We suggest that computations from both domains are necessary for adults and infants to make sense of these events.

Because agents have physical bodies, exist in a physical world, and their plans reflect information about that world, adults’ and infants’ understanding of even simple actions may require the integration of computations between physical and psychological domains. For example, representing the efficiency of an action may require first representing the agent and obstacle as solid bodies, and the agent as a body that can generate force against gravity. The best computational models of how infants understand other people’s goal-directed actions contain a joint model for action planning and physical simulation (Shu et al., 2021). While we have followed a long tradition, from both cognitive neuroscience and developmental psychology, of studying intuitive psychology and physics as contrasting domains, our imposed labels may be obscuring common or linked representations that organize the functions of domain-specific regions like the STS and SMG. Future work could explicitly link the representations from computational models of early intuitive psychology and physics to neural responses to better understand our capacity to reason about agents, acting in a physical world.

### 4.4 Further limitations

In addition to the limitations of this work that we have already discussed above, we would like to mention two others. First, our experiments were devised to test neural responses to VOE events that (by hypothesis) adults and infants had expectations about prior to participating in the study, in just two domains: intuitive physics and intuitive psychology. For now, it is unclear how these results would extend to expectations that participants learn on the fly (Kidd et al., 2012; Saffran et al., 1996), or expectations from other domains, like social relations (Mascaro & Csibra, 2012; Powell & Spelke, 2013; Thomas et al., 2022; Thomsen et al., 2011). Second, we have deliberately studied a narrow subset of the functions of the temporal, parietal, and frontal cortices, in order to test specific hypotheses about the mental processes underlying VOE. For now, it is unknown how portions of the temporal lobes involved in multimodal integration (Campanella & Belin, 2007), portions of the frontal cortex involved in hierarchical control (Badre & Nee, 2018), and portions of the parietal lobes involved in spatial attention (Husain & Nachev, 2007), and additional functions, would respond to these events. However, the methods from this paper could be used to study these topics and regions further.

### 4.5 Conclusion

When an object hovers in midair, or a person acts irrationally, infants look and pay attention to those events. What mental processes account for this behavior: that these events are visually novel, evoke curiosity, and/or violate infants’ expectations about the physical and psychological world? We scanned adults using functional magnetic resonance imaging and found that adults do not merely process such events as novel visual stimuli. Instead, these events evoke distinctively physical and psychological processing, as well as domain-general, internally driven attention. These results serve as a baseline for future studies of infants, and illustrate the promise of using the tools of cognitive neuroscience to address questions about infant minds.

## Supplementary Material

Supplementary Material

## Data Availability

The methods and analyses of these experiments were pre-registered prior to data collection, including several updates. Our pre-registration documents, openly available at https://osf.io/sa7jy/registrations, detail all decisions and updates and the status of data collection and analysis. All experiment scripts, including stimuli shown to participants, as well as the data and analysis scripts required to reproduce statistical results, can be found at https://osf.io/sa7jy/. De-faced brain images from participants in Experiments 1 and 2 who consented to share them (N = 16/17 for Experiment 1; N = 29/32 for Experiment 2) are available on OpenNeuro at https://openneuro.org/datasets/ds004934.

## Footnotes

1
This deviates from our pre-registration which specified 32 scenarios, due to an error in our experimental scripts.

2
We pre-registered this selection procedure but due to an error in this analysis, we originally selected partially different MD focal regions than what is reported in this paper. For full transparency, we report the results from these regions in the SM, Section 5.6.

3
Although we observed habituation of the neural VOE effect in Experiment 1, we still chose to run 4 runs of the VOE task in Experiment 2 because it was unclear whether that habituation occurred because of the repeating stimuli (Experiment 2 had many more stimuli than Experiment 1), and/or because of the repetition of surprising events in general.

4
Originally, we pre-registered this analysis over multivariate effect sizes, rather than univariate effect sizes reported here. However, due to the lack of reliable multivariate information about events, even within domains (despite clear univariate effects), we felt that we could no longer strongly interpret these results. We report the results of the originally pre-registered analysis in the SM.
