# Supplementary material for: Violations of physical and psychological expectations in the human adult brain
Source: Imaging Neurosci (Camb). 2024 Feb 1;2:imag-2-00068. doi: 10.1162/imag_a_00068 (PMC12239824; doi:10.1162/imag_a_00068)
Supplement: Supplementary Material [file imag_a_00068-supp.pdf]

Violations of physical and psychological expectations in the human adult brain

Supplemental Materials

Authors: Shari Liu, Kristen Lydic, Jerry Mei & Rebecca Saxe

For data and code required to reproduce these figures and results, see https://osf.io/sa7jy/. Please direct questions to Shari Liu, at shariliu@jhu.edu.

For additional results, see:
https://rpubs.com/shariliu/nes_exp1_univariate
https://rpubs.com/shariliu/nes_exp1_multivariate
https://rpubs.com/shariliu/nes_exp2_univariate
https://rpubs.com/shariliu/nes_exp2_multivariate

Table of Contents

Supplemental figures.......... 4
1. Behavioral ratings for stimuli.......... 7
2 Preprocessing of neuroimaging data.......... 9
2.1 Experiment 1.......... 9
Anatomical data preprocessing.......... 9
Functional data preprocessing.......... 9
Copyright Waiver.......... 10
2.2 Experiment 2.......... 11
Anatomical data preprocessing.......... 11
Functional data preprocessing.......... 11
Copyright Waiver.......... 13
3. Description of data analysis pipeline.......... 14
Packages and software.......... 14
Processes.......... 14
Convert DICOMs to BIDS.......... 14
Preprocessing.......... 14
Motion exclusions.......... 15
First level analyses.......... 15
Second level analyses.......... 16
Group level analysis.......... 16
4. Procedures for parcel selection and creation.......... 17
4.1 Overview.......... 17
4.2 Details about domain-specific parcel construction.......... 17
5. Validation of localizer tasks.......... 19
6. Additional univariate results.......... 21
6.1 Habituation of the neural VOE signal across runs (manipulation check).......... 21
6.2 Overlap between MD and physics ROIs.......... 21
6.3 Non-focal region univariate results (physics and psychology-action events).......... 22
Event Effects.......... 22
Domain Effects.......... 22
Event x Domain Interactions.......... 23
6.4 Alternative definition for psychology ROIs.......... 23
6.5 Visual statistics.......... 24
6.6 Results from originally selected MD ROIs.......... 26
6.7 Responses to visual novelty in early visual regions.......... 27
6.8 VOE effects for psychology-environment events, all runs.......... 28
6.9 VOE effects by task.......... 29
7. Supplemental MVPA results.......... 32
7.1 Robust univariate, and absent multivariate, event effects.......... 32
7.2 Multivariate region-by-region analysis.......... 33
8. Whole-brain group analyses.......... 36
9. References.......... 39

Supplemental figures

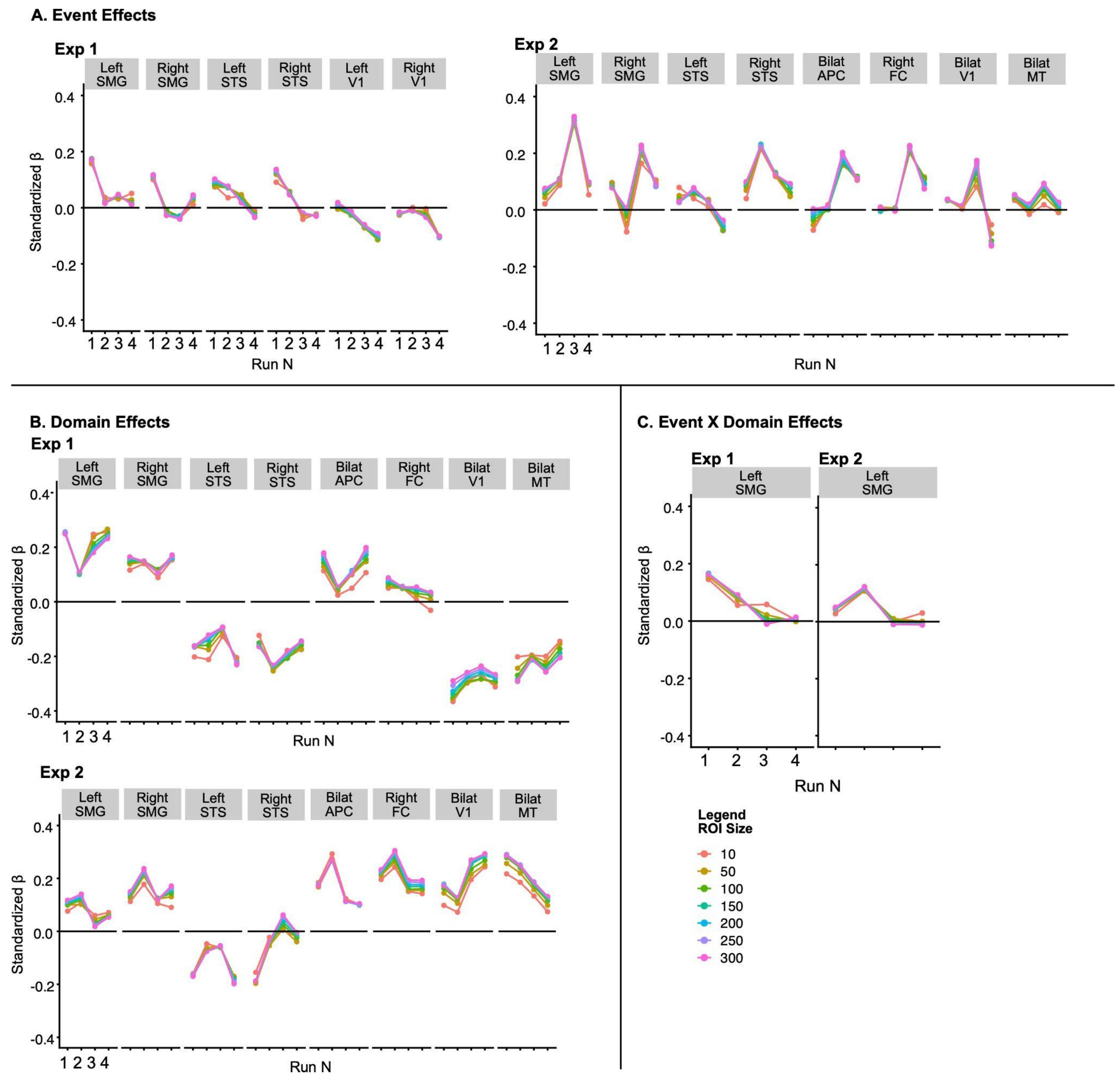

Figure S1. Sensitivity of (A) event effects (unexpected > expected), (B) the domain x event interaction (unexpected > expected, greater for physics than psychology), and (C) domain effects (physics > psychology) across experimental runs and ROI size (10-300 voxels), in the psychology-action and physics events of Experiments 1 and 2. Event effects across runs from bilateral APC and right FC are not shown for Experiment 1, because the VOE data used to choose the ROIs were from runs 2-4, and are thus non-independent from the runs 2-4 results. For all other regions, the data used to select the ROIs were independent of the data extracted from the ROIs.

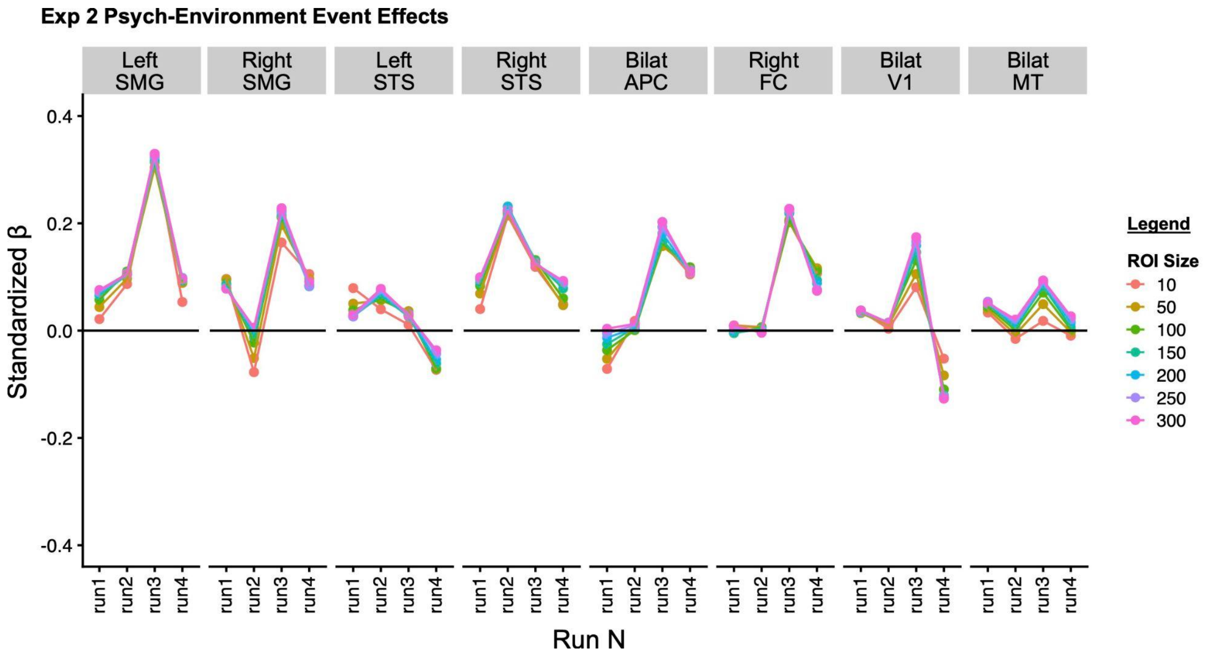

Figure S2. Sensitivity of event effects (unexpected > expected) over runs and ROI size (10-300 voxels) in psychology-environment events of Experiment 2.

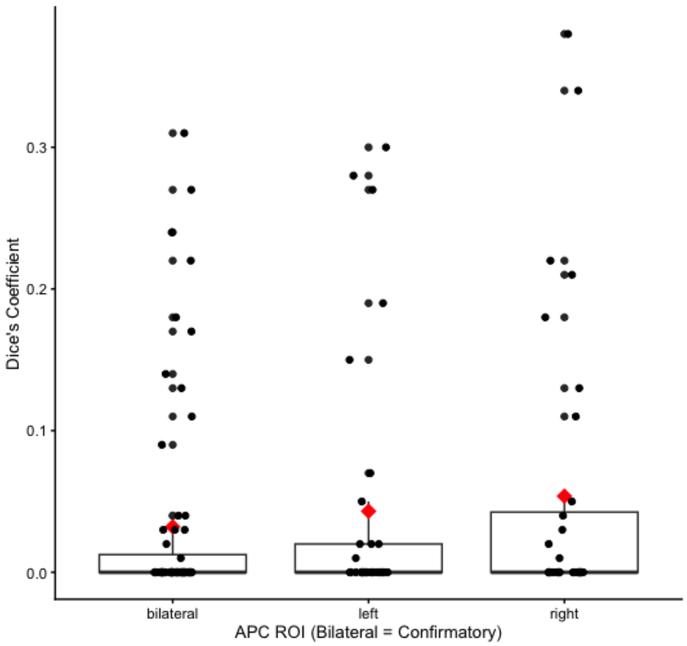

Figure S3. Dice's Coefficient (DC) between each subject's APC ROI and SMG ROIs from Experiment 2. The leftmost boxplot shows DC between bilateral APC and left and right SMG, our pre-registered ROIs. The remaining boxplots show DC between the left SMG and left APC (center), and between right SMG and right APC (right). The median DC for all three plots is 0. The mean DC is plotted in red (< .1 for all ROIs and < .05 for the pre-registered bilateral APC ROI).

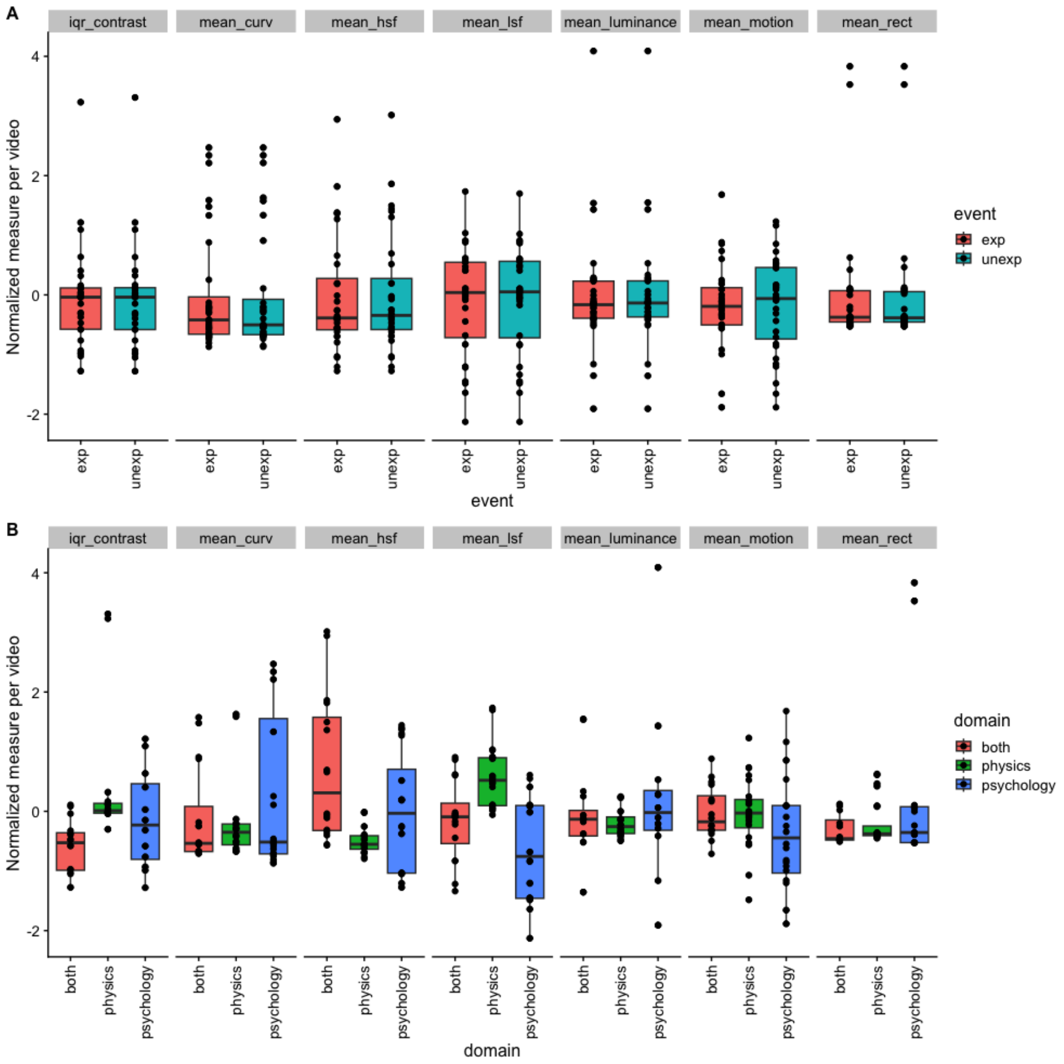

Figure S4. Boxplot of stimulus features, normalized across all videos per feature, (A) for each event type, and (B) for each domain. Each dot represents one video. Panels from left to right: stimulus contrast, curvilinearity, high spatial frequency, low spatial frequency, luminance, motion, rectilinearity.

1. Behavioral ratings for stimuli

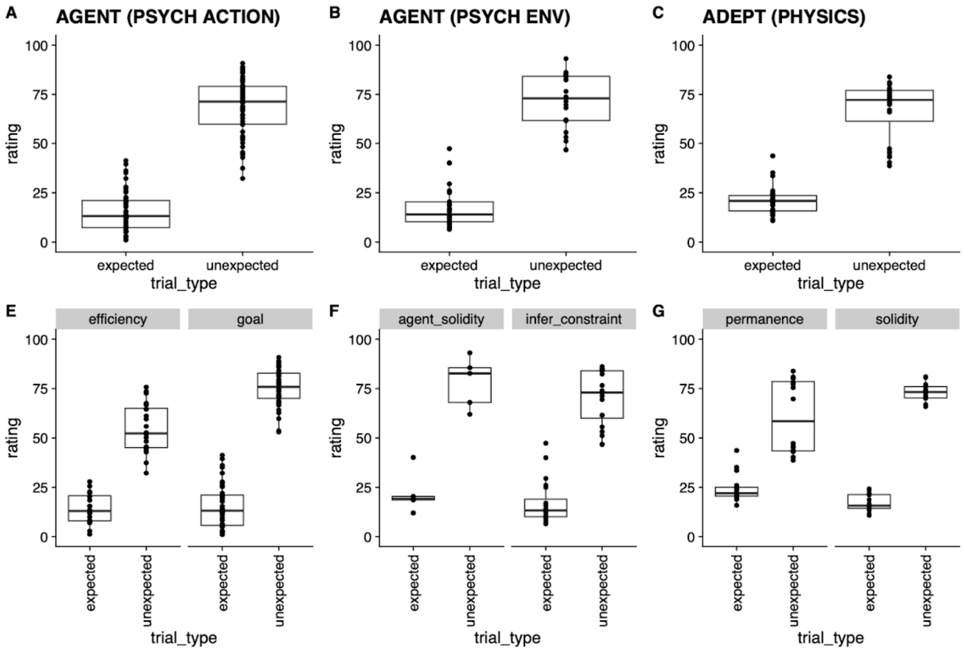

D

	task_domain	cohens_d
1	psych_action	4.81710900365047
2	psych_environment	4.51546226818403
3	physics	4.12245312910946

H

	task_domain	cohens_d
1	efficiency	6.64348083359525
2	goal	5.94517946267836
3	infer_constraint	4.35641479845473
4	agent_solidity	4.35641479845473
5	permanence	2.6915098335523
6	solidity	13.5333615864725

Figure S5. Behavioral ratings ("How surprising?" 0: "Not at all" to 100: "Extremely") from test events in the AGENT (A-B, E-F) and ADEPT (C, G) datasets. Each dot indicates the average behavioral rating per scenario, rated by 8-10 people, grouped by domain (top row) or task (bottom row). Separate groups of people rated expected and unexpected test events from each scenario. (D-H) Effect sizes for the VOE effect (unexpected vs expected ratings), per task (H), and per domain (D).

In prior research, Smith et al (2019, ADEPT) and Shu et al. (2021, AGENT) showed adult participants (Smith et al. N = 60 total, 8 ratings per scenario; Shu et al. N = 200 total, 10 ratings per scenario) a large set of procedurally generated videos based on behavioral infant studies, a subset of which we scanned in the current paper. In these behavioral studies, adult participants saw a familiarization and test event (combined into a single event, for Smith et al.; shown as two separate events, for Shu et al), and rated how surprising each test event was on a scale of 0 to 100, with 0 indicating "not at all surprising" and 100 indicating "extremely surprising", with pairs of events presented in shuffled order, just like in our fMRI experiment.

In these behavioral studies, people from the behavioral studies never saw expected and unexpected outcomes from the same scenario. Furthermore, people saw *only* physics videos (ADEPT dataset), or *only* psychology-action and psychology-environment videos (AGENT data); these two datasets were collected separately. In our fMRI experiment, participants saw trials from both datasets, and saw both outcomes for each scenario, either immediately following each other (Exp 1), or in a separate run of the experiment (Exp 2).

Given the average ratings for each video scenario, we computed an effect size for the VOE effect (unexpected vs expected ratings) for each domain and task. We found large (d > 2) behavioral VOE effects for all tasks and domains. Notably, stimuli from the three domains were rated around equally surprising, with similar effect sizes; this suggests

that any difference in neural VOE responses between domains cannot merely be explained by aggregate differences in how surprising events were across domains.

2 Preprocessing of neuroimaging data

2.1 Experiment 1

Results included in this manuscript come from preprocessing performed using *fMRIprep* 1.2.6 (Esteban, Markiewicz, et al. (2018); Esteban, Blair, et al. (2018); RRID:SCR_016216), which is based on *Nipype* 1.1.7 (Gorgolewski et al. (2011); Gorgolewski et al. (2018); RRID:SCR_002502).

Anatomical data preprocessing

The T1-weighted (T1w) image was corrected for intensity non-uniformity (INU) using `N4BiasFieldCorrection` (Tustison et al. 2010, ANTs 2.2.0), and used as T1w-reference throughout the workflow. The T1w-reference was then skull-stripped using `antsBrainExtraction.sh` (ANTs 2.2.0), using OASIS as target template. Brain surfaces were reconstructed using `recon-all` (FreeSurfer 6.0.1, RRID:SCR_001847, Dale, Fischl, and Sereno 1999), and the brain mask estimated previously was refined with a custom variation of the method to reconcile ANTs-derived and FreeSurfer-derived segmentations of the cortical gray-matter of Mindboggle (RRID:SCR_002438, Klein et al. 2017). Spatial normalization to the ICBM 152 Nonlinear Asymmetrical template version 2009c (Fonov et al. 2009, RRID:SCR_008796) was performed through nonlinear registration with `antsRegistration` (ANTs 2.2.0, RRID:SCR_004757, Avants et al. 2008), using brain-extracted versions of both T1w volume and template. Brain tissue segmentation of cerebrospinal fluid (CSF), white-matter (WM) and gray-matter (GM) was performed on the brain-extracted T1w using `fast` (FSL 5.0.9, RRID:SCR_002823, Zhang, Brady, and Smith 2001).

Functional data preprocessing

For each of the 18 BOLD runs found per subject (across all tasks and sessions), the following preprocessing was performed. First, a reference volume and its skull-stripped version were generated using a custom methodology of *fMRIPrep*. The BOLD reference was then co-registered to the T1w reference using `bbregister` (FreeSurfer) which implements boundary-based registration (Greve and Fischl 2009). Co-registration was configured with nine degrees of freedom to account for distortions remaining in the BOLD reference. Head-motion parameters with respect to the BOLD reference (transformation matrices, and six corresponding rotation and translation parameters) are estimated before any spatiotemporal filtering using `mcflirt` (FSL 5.0.9, Jenkinson et al. 2002). The BOLD time-series, were resampled to surfaces on the following spaces: *fsaverage5*. The BOLD time-series (including slice-timing correction when applied) were resampled onto their original, native space by applying a single, composite transform to correct for head-motion and susceptibility distortions. These resampled BOLD time-series will be referred to as *preprocessed BOLD in original space*, or just *preprocessed BOLD*. First, a reference volume and its skull-stripped version were generated using a custom methodology of *fMRIPrep*. Automatic removal of motion artifacts using independent component analysis (ICA-AROMA, Pruim et al. 2015) was performed on the *preprocessed BOLD on MNI space* time-series after removal of non-steady state volumes and spatial smoothing with an isotropic, Gaussian kernel of 6mm FWHM (full-width half-maximum). Corresponding “non-aggresively” denoised runs were produced after such smoothing. Additionally, the “aggressive” noise-regressors were collected and placed in the corresponding confounds file. The BOLD time-series were resampled to MNI152NLin2009cAsym standard space, generating a *preprocessed BOLD run in MNI152NLin2009cAsym space*. First, a reference volume and its

skull-stripped version were generated using a custom methodology of *fMRIPrep*. Several confounding time-series were calculated based on the *preprocessed BOLD*: framewise displacement (FD), DVARS and three region-wise global signals. FD and DVARS are calculated for each functional run, both using their implementations in *Nipype* (following the definitions by Power et al. 2014). The three global signals are extracted within the CSF, the WM, and the whole-brain masks. Additionally, a set of physiological regressors were extracted to allow for component-based noise correction (*CompCor*, Behzadi et al. 2007). Principal components are estimated after high-pass filtering the *preprocessed BOLD* time-series (using a discrete cosine filter with 128s cut-off) for the two *CompCor* variants: temporal (tCompCor) and anatomical (aCompCor). Six tCompCor components are then calculated from the top 5% variable voxels within a mask covering the subcortical regions. This subcortical mask is obtained by heavily eroding the brain mask, which ensures it does not include cortical GM regions. For aCompCor, six components are calculated within the intersection of the aforementioned mask and the union of CSF and WM masks calculated in T1w space, after their projection to the native space of each functional run (using the inverse BOLD-to-T1w transformation). The head-motion estimates calculated in the correction step were also placed within the corresponding confounds file. All resamplings can be performed with *a single interpolation step* by composing all the pertinent transformations (i.e. head-motion transform matrices, susceptibility distortion correction when available, and co-registrations to anatomical and template spaces). Gridded (volumetric) resamplings were performed using `antsApplyTransforms` (ANTs), configured with Lanczos interpolation to minimize the smoothing effects of other kernels (Lanczos 1964). Non-gridded (surface) resamplings were performed using `mri_vol2surf` (FreeSurfer).

Many internal operations of *fMRIPrep* use *Nilearn* 0.5.0 (Abraham et al. 2014, RRID:SCR_001362), mostly within the functional processing workflow. For more details of the pipeline, see the section corresponding to workflows in *fMRIPrep*'s documentation.

Copyright Waiver

The above boilerplate text was automatically generated by fMRIPrep with the express intention that users should copy and paste this text into their manuscripts unchanged. It is released under the CC0 license.

2.2 Experiment 2

Results included in this manuscript come from preprocessing performed using fMRIPrep 2.0.2 (Esteban, Markiewicz, et al. (2018); Esteban, Blair, et al. (2018); RRID:SCR_016216), which is based on Nipype 1.8.5 (K. Gorgolewski et al. (2011); K. J. Gorgolewski et al. (2018); RRID:SCR_002502).

Anatomical data preprocessing

A total of 1 T1-weighted (T1w) images were found within the input BIDS dataset.The T1-weighted (T1w) image was corrected for intensity non-uniformity (INU) with `N4BiasFieldCorrection` (Tustison et al. 2010), distributed with ANTs 2.3.3 (Avants et al. 2008, RRID:SCR_004757), and used as T1w-reference throughout the workflow. The T1w-reference was then skull-stripped with a *Nipype* implementation of the `antsBrainExtraction.sh` workflow (from ANTs), using OASIS30ANTs as target template. Brain tissue segmentation of cerebrospinal fluid (CSF), white-matter (WM) and gray-matter (GM) was performed on the brain-extracted T1w using `fast` (FSL 6.0.5.1:57b01774, RRID:SCR_002823, Zhang, Brady, and Smith 2001). Brain surfaces were reconstructed using `recon-all` (FreeSurfer 7.2.0, RRID:SCR_001847, Dale, Fischl, and Sereno 1999), and the brain mask estimated previously was refined with a custom variation of the method to reconcile ANTs-derived and FreeSurfer-derived segmentations of the cortical gray-matter of Mindboggle (RRID:SCR_002438, Klein et al. 2017). Volume-based spatial normalization to two standard spaces (MNI152NLin2009cAsym, MNI152NLin6Asym) was performed through nonlinear registration with `antsRegistration` (ANTs 2.3.3), using brain-extracted versions of both T1w reference and the T1w template. The following templates were selected for spatial normalization: *ICBM 152 Nonlinear Asymmetrical template version 2009c* [Fonov et al. (2009), RRID:SCR_008796; TemplateFlow ID: MNI152NLin2009cAsym], *FSL's MNI ICBM 152 non-linear 6th Generation Asymmetric Average Brain Stereotaxic Registration Model* [Evans et al. (2012), RRID:SCR_002823; TemplateFlow ID: MNI152NLin6Asym].

Functional data preprocessing

For each of the 10 BOLD runs found per subject (across all tasks and sessions), the following preprocessing was performed. First, a reference volume and its skull-stripped version were generated using a custom methodology of *fMRIPrep*. Head-motion parameters with respect to the BOLD reference (transformation matrices, and six corresponding rotation and translation parameters) are estimated before any spatiotemporal filtering using `mcflirt` (FSL 6.0.5.1:57b01774, Jenkinson et al. 2002). BOLD runs were slice-time corrected to 0.95s (0.5 of slice acquisition range 0s-1.9s) using `3dTshift` from AFNI (Cox and Hyde 1997, RRID:SCR_005927). The BOLD time-series (including slice-timing correction when applied) were resampled onto their original, native space by applying the transforms to correct for head-motion. These resampled BOLD time-series will be referred to as *preprocessed BOLD in original space*, or just *preprocessed BOLD*. The BOLD reference was then co-registered to the T1w reference using `bbregister` (FreeSurfer) which implements boundary-based registration (Greve and Fischl 2009). Co-registration was configured with six degrees of freedom. Several confounding time-series were calculated based on the *preprocessed BOLD*: framewise displacement (FD), DVARS and three region-wise global signals. FD was computed using two formulations following Power (absolute sum of relative motions, Power et al. (2014)) and Jenkinson (relative root mean square displacement

between affines, Jenkinson et al. (2002)). FD and DVARS are calculated for each functional run, both using their implementations in *Nipype* (following the definitions by Power et al. 2014). The three global signals are extracted within the CSF, the WM, and the whole-brain masks. Additionally, a set of physiological regressors were extracted to allow for component-based noise correction (*CompCor*, Behzadi et al. 2007). Principal components are estimated after high-pass filtering the *preprocessed BOLD* time-series (using a discrete cosine filter with 128s cut-off) for the two *CompCor* variants: temporal (tCompCor) and anatomical (aCompCor). tCompCor components are then calculated from the top 2% variable voxels within the brain mask. For aCompCor, three probabilistic masks (CSF, WM and combined CSF+WM) are generated in anatomical space. The implementation differs from that of Behzadi et al. in that instead of eroding the masks by 2 pixels on BOLD space, a mask of pixels that likely contain a volume fraction of GM is subtracted from the aCompCor masks. This mask is obtained by dilating a GM mask extracted from the FreeSurfer's *aseg* segmentation, and it ensures components are not extracted from voxels containing a minimal fraction of GM. Finally, these masks are resampled into BOLD space and binarized by thresholding at 0.99 (as in the original implementation). Components are also calculated separately within the WM and CSF masks. For each CompCor decomposition, the *k* components with the largest singular values are retained, such that the retained components' time series are sufficient to explain 50 percent of variance across the nuisance mask (CSF, WM, combined, or temporal). The remaining components are dropped from consideration. The head-motion estimates calculated in the correction step were also placed within the corresponding confounds file. The confound time series derived from head motion estimates and global signals were expanded with the inclusion of temporal derivatives and quadratic terms for each (Satterthwaite et al. 2013). Frames that exceeded a threshold of 0.5 mm FD or 1.5 standardized DVARS were annotated as motion outliers. Additional nuisance timeseries are calculated by means of principal components analysis of the signal found within a thin band (*crown*) of voxels around the edge of the brain, as proposed by (Patriat, Reynolds, and Birn 2017). The BOLD time-series were resampled into standard space, generating a *preprocessed BOLD run in MNI152NLin2009cAsym space*. First, a reference volume and its skull-stripped version were generated using a custom methodology of *fMRIPrep*. The BOLD time-series were resampled onto the following surfaces (FreeSurfer reconstruction nomenclature): *fsaverage*. Automatic removal of motion artifacts using independent component analysis (ICA-AROMA, Pruim et al. 2015) was performed on the *preprocessed BOLD on MNI space* time-series after removal of non-steady state volumes and spatial smoothing with an isotropic, Gaussian kernel of 6mm FWHM (full-width half-maximum). Corresponding “non-aggresively” denoised runs were produced after such smoothing. Additionally, the “aggressive” noise-regressors were collected and placed in the corresponding confounds file. *Grayordinates* files (Glasser et al. 2013) containing 91k samples were also generated using the highest-resolution `fsaverage` as intermediate standardized surface space. All resamplings can be performed with *a single interpolation step* by composing all the pertinent transformations (i.e. head-motion transform matrices, susceptibility distortion correction when available, and co-registrations to anatomical and output spaces). Gridded (volumetric) resamplings were performed using `antsApplyTransforms` (ANTs), configured with Lanczos interpolation to minimize the smoothing effects of other kernels (Lanczos 1964). Non-gridded (surface) resamplings were performed using `mri_vol2surf` (FreeSurfer).

Many internal operations of *fMRIPrep* use *Nilearn* 0.9.1 (Abraham et al. 2014, RRID:SCR_001362), mostly within the functional processing workflow. For more details of the pipeline, see the section corresponding to workflows in *fMRIPrep*'s documentation.

Copyright Waiver

The above boilerplate text was automatically generated by fMRIPrep with the express intention that users should copy and paste this text into their manuscripts unchanged. It is released under the CC0 license.

3. Description of data analysis pipeline

All analyses, after preprocessing, used the standard fMRI pipeline from the MIT Saxe Lab (https://saxelab.mit.edu/).

Packages and software
We use singularity containers for all processes in the lab pipeline. We are currently working on making our containers accessible publicly, but for now here is a list of software versions we use:

- Singularity: 3.4.1
- Docker image version of singularity available here
- We also use FSL for group level analyses:
  - FSL (for randomise, cluster): 5.0.9
  - Docker image available here

Software below used within Singularity containers:
- heudiconv: 0.9.0 (data analyzed before summer 2022: 0.5.4.dev1)- singularity image from Docker here
- fmriprep: 22.0.2 (data analyzed before summer 2022: v1.2.6)- singularity image from Docker here
- nipy/nipype: 1.5.1 - singularity image from Docker (closest match) here

Conda:
- 4.5.12 (heudiconv; fmriprep)
- 4.8.4 (nipype; univariate/multivariate ROI analyses)
- Python:
  - 3.6.7 (used in heudiconv container)
  - 3.7.1 (fmriprep container)
  - 3.6.5 (nipype container)
  - 3.8.3 (univariate/multivariate ROI analyses container)

Processes
Convert DICOMs to BIDS
We use heudiconv in a singularity container to convert fMRI data to BIDS format based on the experimental design.

Preprocessing
We preprocess data using fMRI data using the fMRIprep toolbox within a singularity container. Here is an example of how we call fMRIprep using the standard flags for our pipeline:

```
fmriprep $data_directory/BIDS $data_directory/BIDS/derivatives participant
--participant_label $subject_id --mem_mb 15000 --ignore slicetiming
--use-aroma -w $scratch --fs-license-file $FSL_license_path --output-spaces
MNI152NLin6Asym:res-2
```

Note, fMRIprep is technically nondeterministic; there is slight computational variability that results in slightly different reconstructions each time fMRIprep is run. For this

reason, we try to maintain a standard of sharing subject-level preprocessed data as well as raw BIDS data when possible (i.e., when we have consent to share).

fMRIprep includes standard fMRI preprocessing, and with the `--use-aroma` flag, it also runs "soft" artifact correction and generates the confounds used as nuisance regressors in first-level modeling. See fMRIprep pages (linked above) for details.

Motion exclusions

After preprocessing, we use fMRIprep's Frame Displacement (FD) estimate per run to flag volumes within each run that have greater than X units of change (typically: 0.4 units) in FD from the start of the run. These volumes are excluded from first-level analyses. If greater than Y% (typically: 25%) of any run is flagged as motion, the whole run is excluded.

First level analyses

We use Nipype to combine tools from different software packages, mainly relying on Nipype's FSL interface to fit the run-level (first-level) GLM. The model is fit using FSL's FEAT tool.

There are event regressors per each contrast specified in the study-specific contrast file, as well as confounds imported from fMRIprep preprocessing. Specifically, this step relies on the confounds text file that fmriprep outputs and the realigned and normalized bold and anatomical images, as well as the events.tsv files located inside the BIDS directory specifying the onset and duration for every condition in the experiment (instructions to create the events.tsv file below).

The design for the experiment is calculated from those event files, along with nuisance regressors specified below. Each event regressor is convolved with a double-gamma HRF, and a high-pass filter is applied to both the data and the model.

Artifact detection is performed using nipype’s RapidART toolbox, which is itself an implementation of SPM’s ART toolbox. Individual TRs are identified as outliers if they exceed a motion threshold of more than .4 units of frame displacement, or if the average signal intensity of that volume is more than three standard deviations away from the mean average signal intensity.

In addition to the ART outliers (one regressor per outlier volume), the current Saxelab script includes a summary movement regressor (framewise displacement, or FD), and 6 anatomical CompCor regressors that are intended to control for the average signal in white matter and CSF. All regressors other than head movement parameters were convolved with a standard double-gamma hemodynamic response function, with a high pass filter of 1/210 Hz (Experiment 1) or 1/229 Hz (Experiment 2) applied to both the data and the model. Event regressors were defined as a boxcar from the start and end of each block (localizer tasks) or event (VOE task).

A smoothing kernel of 6mm is applied to the preprocessed bold images, and finally, FSL’s GLM runs the first-level model. The current default is to run the model in MNI space.

Contrasts are estimated based on the contrasts specified in the contrasts.tsv file, located in the data/BIDS/code directory.

The standard outputs of an FSL analysis are created in the output directory, including parameter estimates (pe.nii.gz), contrast estimates (con.nii.gz), and residuals. For exploring significance at the run level, the con*zstat.nii.gz are the most useful files, while higher-level models will use the cope and varcope images as inputs to their mixed-effects models.

Second level analyses
Subject-level or second-level modeling combines the GLMs across runs, per subject.

The subject-level scripts will take the data from first level analyses and do operations on them; namely, we use the copes (beta estimates) and varcopes (variance estimates) using FSL's fixed-effects flow. We again use Nipype to execute this. Specifically, we use the FSL FEAT sub-tool called FLAME (FMRIB's Local Analysis of Mixed Effects).

There are two avenues for combining run-level model outputs after creating first-level models, though the second is more commonly used, and also includes the outputs of the first:

(1) Traditional: Create a single second-level model combining all runs of a task, per subject and per contrast.

(2) Iterative: Iteratively create a second-level model for each set of n-1 runs (excluding 1 run from all n runs), per task, per subject, per contrast. (Note: we will call each of these leave-one-run-out combinations a "fold.") This allows us to e.g., select the top voxels based on n-1/n of the data and extract the betas only from the held-out run. We repeat for each possible fold (leave out each run once), then average the results from the held-out runs.

Group level analysis
During second-level modeling, we created one model for each task (VOE, DOTSloc, spWMloc, motionLoc) for each participant. These maps were then passed to group-level modeling, wherein for each contrast, across subjects, we used FSL's RANDOMISE to perform a nonparametric one-sample t-test of the contrast values across subjects against 0, with 5000 permutations, in MNI space, with a threshold of alpha = .05, FWE-corrected, using threshold-free cluster enhancement (TFCE). In Experiment 1, we used variance smoothing, σ=6mm, following the recommendation of (Nichols & Holmes, 2002), due to its small sample size.

4. Procedures for parcel selection and creation

4.1 Overview

We aimed to identify the neural correlates of each of the hypothesized cognitive processes that underlie the VOE effect: domain-specific psychological or physical processing, and domain-general early visual processing and/or goal-directed attention. This section describes how we chose parcels, or search spaces for subject-specific fROI definition. In total, we studied responses in 42 parcels across the cortex. We chose parcels for psychology and physics regions from a combination of prior literature, and exploratory analyses on group data from Experiment 1 that were independent of the functional data we analyzed from the VOE task (selected using runs 2-4, and used to study the responses in run 1). The 18 non-overlapping domain-specific parcels (search spaces) we created from independent data spanned regions previously implicated in theory of mind, action understanding, and physical reasoning, as well as regions in the ventral and lateral occipital cortices and parahippocampal gyrus. The 24 non-overlapping domain-general parcels came from prior work: 4 early visual regions (parcels from Pramod et al., 2022 (Pramod et al., 2022), and 20 regions from the multiple demand network (parcels from https://evlab.mit.edu/funcloc/). The early visual parcels were derived from the Desikan-Killiany and Destrieux cortical parcellations in Freesurfer, and the multiple demand parcels were created based on functional data from a probabilistic overlap map from 197 adult participants who performed a spatial working memory task (the same task we scanned, spWMloc). These regions were selected prior to data collection for Experiment 2.

4.2 Details about domain-specific parcel construction

Our domain-specific parcels were derived from group-level data on the DOTSloc task, group-level data from runs 2-4 of the VOE task (with the held-out run 1 reserved for studying the VOE response), and parcels from Pramod et al (2022) of the frontoparietal physics regions, which respond more during judgments of the physical stability of block towers than judgments of the color of the blocks in the same stimuli. First, we created p maps with a relaxed threshold of $p = 0.2$ (TCFE) for both the DOTSloc and VOE data, for the contrasts social >< physical. Then, we found intersecting voxels between (i) the p map for the physical > social contrast in the DOTSloc task and (ii) and the frontoparietal map from Pramod et al. (2022). Next, we found intersecting voxels between the p map for the physical > social contrast from the (i) DOTSloc task, and (ii) the VOE task. Lastly, we found intersecting voxels between the p map for the social > physical contrast from (i) the DOTSloc task and (ii) the VOE task. We dropped clusters that were redundant across these intersection maps or appeared in the cerebellum, flipped the parcel for left SMG over to the right hemisphere to make a right SMG parcel, and combined small clusters together. Finally, we inflated the parcels to make a generous search space, checked for intersections between parcels and removed overlapping voxels and masked the resulting parcels with an MNI brain mask for each hemisphere to ensure clean separation.

In the end, we created 4 physical clusters that were derived from an intersection of the DOTSloc and frontoparietal parcels, 4 physical clusters that were derived from an intersection of the DOTSloc and VOE tasks (physical > social), and 10 social clusters that were derived from an intersection of the DOTSloc and VOE tasks (social > physical). All of these masks were fixed before data collection in Experiment 2, and are

openly available at
https://osf.io/ryzq5/?view_only=1a8d9a4d1c8c4aa2b8bc929c9653a9ab.

5. Validation of localizer tasks

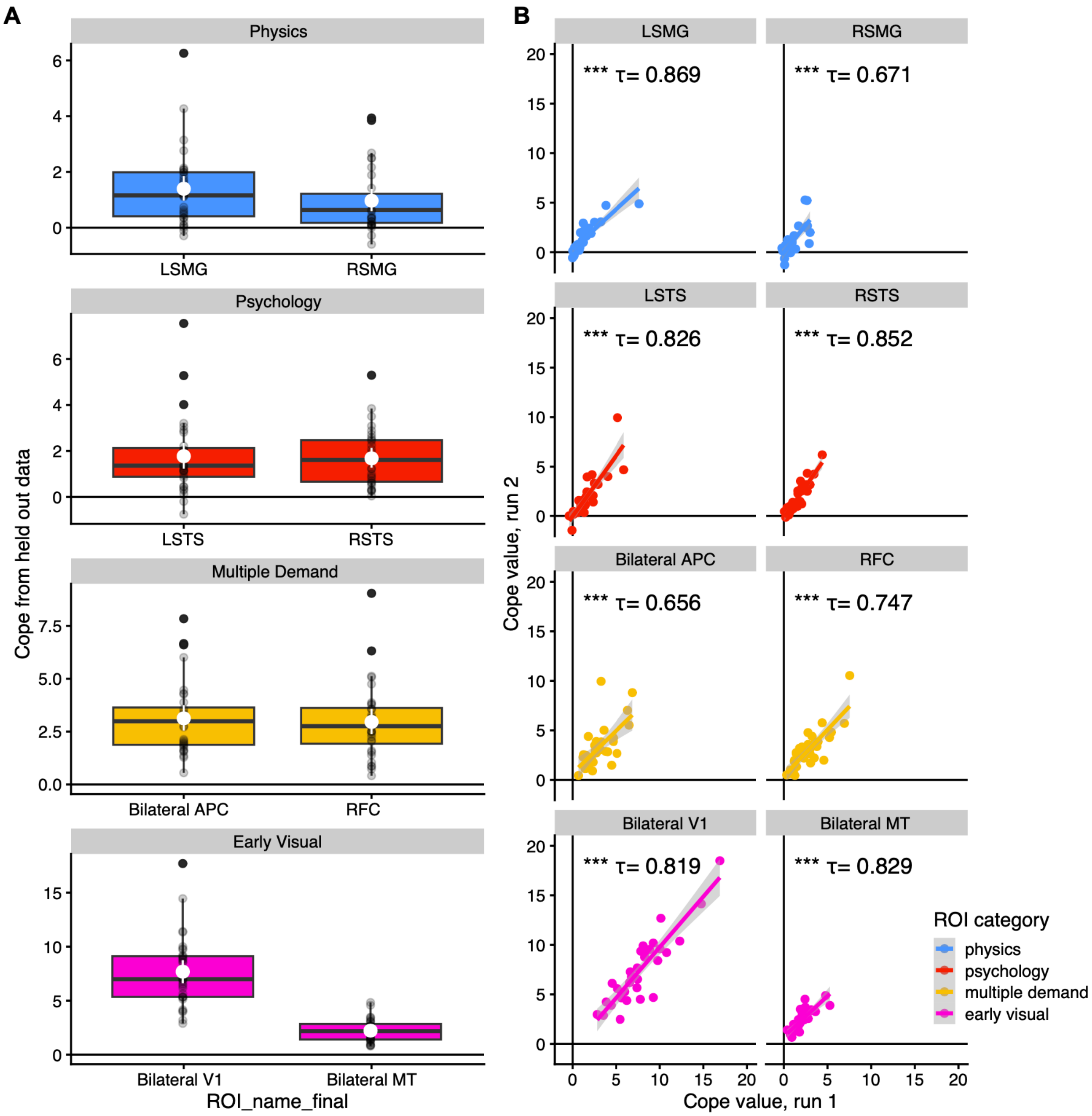

Figure S6. Results of validation analysis over localizer tasks. (A) Responses to localization contrast from opposite runs, averaged per subject (for physics ROIs, physical > social in DOTSloc; for psychology ROIs, social > physical in DOTSloc; for MD ROIs, hard > easy in spWMloc; for bilateral V1, stimuli > rest in spWMloc; for bilateral MT, coherent > incoherent motion in motionLoc). White dots and error bars indicate means and bootstrapped 95% confidence intervals. Note that axes differ between panels. (B) Split-half reliability, measured using Kendall's tau between held out responses in both runs, including a Spearman-Brown correction, $2\tau/(1+\tau)$. ~ $p < .10$, * $p < .05$, ** $p < .01$, *** $p < .001$, one-tailed.

We validated our localizer tasks from Experiment 2 in two ways. First, we conducted a leave-one-run-out analysis, asking whether the focal ROIs identified in individual

subjects using the localizer contrasts, listed in Figure 2B, responded to the same contrasts in held out data from the same task. We took first-level maps (per run, per subject), and for all subjects who contributed data from two runs, (1) selected ROIs using the top 100 method as described in the main text using one run, and (2) extracted their average responses in the other run, using the same contrast. We did this twice per subject (run 1 for ROI selection, run 2 for ROI extraction, and vice versa), and averaged the resulting held out responses per subject. These responses are plotted in Figure S6A; in all ROIs, the response was reliably greater than 0.

Second, we took the extracted ROI responses in each of the two runs, and related them to each other using Kendall’s tau, with the alternative hypothesis that the relationship between paired responses across runs is larger than 0. We chose this metric because of the non-normal distribution of responses, as well as this measure’s robustness to outliers. We also applied a Spearman-Brown correction to convert this into an estimate of the equivalent level of reliability for the full (non-split) data. These values are plotted in Figure S6B; in all ROIs, split half reliability was high (τ range: 0.656-0.869).

6. Additional univariate results

All univariate analyses were carried out using packages lme4 (Bates et al. 2015), lmerTest (Kuznetsova et al. 2017), and lsmeans (Lenth, 2016).

6.1 Habituation of the neural VOE signal across runs (manipulation check)

In Experiment 1, we checked whether the size of the VOE effect declines over runs, in left and right SMG and STS, where we predicted we would find domain-specific effects. The two MD regions were excluded from this analysis because the data used to identify them, from runs 2-4, is non-independent of the data for this analysis. We fit a linear mixed effects model including the interaction between run number and event as fixed effects, and subject ID as a random intercept (formula: `meanbeta ~ extracted_run_number * event + (1|subjectID)`). We then extracted the main effect of event per run using `lsmeans()`. We found that whereas there was a significant VOE effect in run 1 (B = 0.455, p = <.001, two-tailed), this effect was absent in the other runs (run 2: B = 0.13, p = 0.247, two-tailed; run 3: B = 0.019, p = 0.865, two-tailed; run 4: B = 0.002, p = 0.989, two-tailed). Thus, we proceeded with our ssfROI data from just the first VOE run, and pre-registered this analysis procedure as a way to select between including data from all runs, or just the first 2 runs, in Experiment 2.

In Experiment 2, this event by run manipulation check was conducted in all regions for which we predicted a positive effect (left and right SMG, left and right STS, bilateral APC, right FC). Similarly to Experiment 1, we found that there were marginal or significant event effects in runs 1 and 2 (run 1: B = -0.298, p = 0.069, two-tailed; run 2: B = -0.328, p = 0.045, two-tailed), but no significant event effects in runs 3 or 4 (run 3: B = -0.054, p = 0.744, two-tailed; run 4: B = -0.066, p = 0.685, two-tailed). Thus we followed our plan to restrict all subsequent confirmatory analyses to the first two runs, using the same set of mixed effects models and significance thresholds as for Experiment 1.

For the exploratory, psychology-environment events, we again checked whether the VOE effect declined across all runs in the same regions as for the psychology-action and physics events. Unlike the VOE effects from the physics and psychology-action events, the VOE effects we explored from this stimulus set did not habituate over runs (run 1: B = 0.23, p = 0.388, two-tailed), this effect was absent in the other runs (run 2: B = 0.286, p = 0.284, two-tailed; run 3: B = 0.915, p = 0.001, two-tailed; run 4: B = 0.308, p = 0.248, two-tailed). Based on these considerations, in the main text, we presented the results from these events in the same portion of the data as our primary analysis (runs 1 and 2), and from all available data (runs 1-4). Model formula: `meanbeta ~ event + (1|subjectID)`.

See Figures S1-2 for visualizations of effect sizes across runs in both experiments.

6.2 Overlap between MD and physics ROIs

The search space for a focal multiple demand ROI, the bilateral anterior parietal cortex (APC), substantially overlapped with the search spaces for 2 physical ROIs, the left and right supramarginal gyrus (SMG). How much do the ssfROIs, which were defined for each subject based either on a working memory task (spWMloc) or a physical prediction task (DOTSloc), overlap with each other? To investigate this question, we computed

Dice's Coefficient (DC; Bennett & Miller, 2010) between the APC and left/right SMG ROIs for each subject, which expresses the amount of spatial overlap between the two regions: $DC(X, Y)= 2(X\cap Y)/(X+Y)$, where X+Y is the total number of voxels across the regions X and Y (in our case, 200 voxels, 100 per ROI), and $X\cap Y$ is the number of voxels that occupy the same location. We found that the median Dice's coefficient between each of the SMG ROIs and the APC ROIs was 0 (LSMG range: 0-0.3; RSMG range: 0-0.38). For the majority of participants (21/32 for LSMG; 20/32 for RSMG), there was no overlap between voxels most selective for physical reasoning, and those most selective for attentional demand. See Figure S3.

6.3 Non-focal region univariate results (physics and psychology-action events)
In Experiments 1 and 2, we tested for VOE effects (unexpected > expected) for physics and psychology-action events across a larger set of domain-specific parcels we made based on independent data from Experiment 1. Parcels from prior work on physical reasoning (Pramod et al., 2022) and attentional demand (Fedorenko et al., 2013), using a Bonferroni corrected alpha threshold of $p = .05/24 = .002$ for domain-general regions (24 total), and of $p = .05/18 = .003$ for domain-specific regions (18 total).

Event Effects
No regions beyond our focal regions in Experiment 1 or 2 showed a main effect of event that passed these stringent significance thresholds. The one region that passed this threshold overlapped substantially with our RFC ROI. See https://rpubs.com/shariliu/nes_results, Section 5.1.1, for results from all regions.

Domain Effects
In both Experiments 1 and 2, both domain-specific and domain-general regions showed a greater response for physical than psychological events, or vice versa, that met our stringent significance threshold. See https://rpubs.com/shariliu/nes_results, Section 5, for results from all regions.

In Experiment 1, the regions that responded more to physical events were:

- Left and right visual medial cortex (physics regions): Left [0.64,1.06], B=0.85, p<.001, two-tailed, BF > 1000; right [0.355,0.733], B=0.544, p<.001, two-tailed, BF > 1000
- Left and right anterior parietal cortex (physics regions; both are part of our combined bilateral APC): Left [0.075,0.33], B=0.202, p=0.002, two-tailed, BF = 1.114; right `r [0.075,0.33], B=0.202, p=0.002, two-tailed, BF = 1.114

In Experiment 1, the regions that responded more to psychological events were:

- Left and right lateral and ventral visual cortex. Left: [-0.715, -0.369], B = -0.542, p < .001, two-tailed, BF > 1000. Right: [-0.523, -0.168], B = -0.346, p < .001, two-tailed, BF = 16.472

In Experiment 2, the physics ROIs with the biggest univariate preference for physical events (by effect size) were:

- Right medial visual cortex, [0.894,1.241], B=1.068, p<.001, two-tailed,BF > 1000
- Left medial visual cortex, [0.704,1.11], B=0.907, p<.001, two-tailed, BF > 1000
- Right superior parietal cortex, [0.671,1.175], B=0.923, p<.001, two-tailed, BF > 1000

In Experiment 2, the MD/early visual ROIs with the biggest univariate preference for physical events (by effect size) were:

- Right MT, [0.611,0.877], B=0.744, p<.001, two-tailed, BF > 1000
- Right posterior parietal cortex, [0.879,1.434], B=1.157, p<.001, two-tailed, BF > 1000
- Right mid parietal cortex, [0.66,1.151], B=0.905, p<.001, two-tailed, BF > 1000

In Experiment 2, two regions showed a greater response to psychological than physical events. Both were psychology ROIs:

- Left lateral and ventral visual cortex, [-0.768,-0.396], B=-0.582, p<.001, two-tailed, BF > 1000
- Left MPFC, [-0.787,-0.275], B=-0.531, p<.001, two-tailed, BF = 56.18

Event x Domain Interactions

No non-focal regions showed an event x domain interaction, even by the more lenient p < .05 threshold. See https://rpubs.com/shariliu/nes_results, Section 5.1.1, for results from all regions.

In sum, like in our confirmatory results, we found strong evidence for domain-specific responses, but weaker evidence for event-driven responses, in both ROIs that we selected and defined to be domain-specific and domain-general.

6.4 Alternative definition for psychology ROIs

Why did we fail to observe a consistent main effect of event, or a VOE effect only for psychological events, in left and right STS?

One possibility is a conceptual mismatch between the social information evoked by our independent localizer (two agents interacting socially), and the social information evoked by the VOE task (a single agent acting to achieve a physical goal, in the psychology-action events of Experiment 2). To test this possibility, we repeated the univariate analysis in left and right STS, except this time, we selected ssfROIs in STS based on the psychological > physical contrast from an independent split of the VOE task (runs 3-4 in Experiment 2; top 100 voxels by the z statistic like other analyses).

In Experiment 2, we found that left and right STS, defined based on a contrast between psychological events (involving instrumental action) and purely physical events, showed a reliable preference for psychological events (left STS: [-0.319,-0.045], B=-0.182, p=0.01, two-tailed, BF = 0.317; right STS: [-0.415,-0.206], B=-0.31, p<.001, two-tailed, BF = 58439.327). However, these left and right STS ROIs did not respond more to unexpected than expected events (left STS: [-0.218,0.055], B=-0.081, p=0.246, two-tailed, BF = 0.022; right STS: [-0.187,0.022], B=-0.082, p=0.125, two-tailed, BF = 0.027). Neither region showed an interaction between domain and event (left STS: [-0.131,0.142], B=0.005, p=0.937, two-tailed, BF = 0.011; right STS: [-0.141,0.069], B=-0.036, p=0.502, two-tailed, BF = 0.011).

In summary, across two ROI definitions,, we did not find evidence for domain-general or domain-specific prediction error in “psychology” STS ROIs, though STS did respond more to psychological than physical events, characteristic of its social functions.

6.5 Visual statistics

We tested for the robustness of our VOE effects (domain-specific event response in SMG, domain-general event responses in APC and RFC) accounting for the variability in the lower-level visual statistics in our stimuli. We conducted this exploratory analysis on the data from Experiment 2, which contained many more scenarios than Experiment 1, to maximize sensitivity to stimulus-driven effects.

For each video, we calculated the amount of luminance, contrast, motion, high spatial frequency info, low spatial frequency info, curvilinearity, and rectilinearity, z-scored across videos per feature.

To calculate spatial frequency, we computed Fourier transform on each frame of each video, and followed methods and cut-offs from Rajimehr et al. (2011) to calculate high and low spatial frequency per frame. To calculate rectilinearity, we applied angled Gabor filters (90° and 180°) with four different spatial frequencies (1, 2, 4, and 8) to each pixel. Averages were taken per frame of each stimulus video, and across frames per video. To calculate curvilinearity, we used a similar method using angled Gabor filters (30°, 60°, 90°, 120°, 150°, and 180°) with five different curve depths. These methods followed Kosakowski et al. (2022). To calculate luminance, we split each frame of each stimulus video into separate R, G, and B channels and computed luminance using the following formula: b = (b / 255) * 2.2, g = (g / 255) * 2.2, r = (r / 255) * 2.2; luminance = 0.2126 * r + 0.7152 * g + 0.0722 * b (https://en.wikipedia.org/wiki/Relative_luminance). To calculate the contrast of each video, we converted each frame to grayscale and obtained the interquartile range of grayscale intensity. For all of the above visual features, we calculated each feature per frame and then averaged across frames to obtain a single value per stimulus. Finally, to calculate motion energy, we followed the methods of Nishimoto et al. (2011). We passed each stimulus video through a series of 3D spatiotemporal Gabor wavelet filters to determine the strength of each motion energy direction and speed. Then we calculated the mean value across all filters, resulting in one value per stimulus.

We found that two visual features, high and low spatial frequency, were highly correlated in many of the models including visual features as predictors. Low spatial frequency was excluded from all models to avoid issues of multicollinearity. See SM for full univariate results on psychology-action and physics events from Experiment 2, including these per-video features as regressors.

Then, we built a GLM to estimate one beta per presentation of each video (i.e. 16 familiarization-test pairs, 32 betas per run) and extracted these betas in the same ssfROIs as the confirmatory analysis. Finally, we fit the same mixed effects models as the confirmatory analysis, in the same regions, using these video-specific betas, while also adding in fixed effects for the 7 visual statistics. Low spatial frequency was dropped from all models given high collinearity with high spatial frequency. Model formula: `meanbeta ~ event * domain + normalized_iqr_contrast + normalized_mean_luminance + normalized_mean_motion + normalized_mean_hsf + normalized_mean_rect + normalized_mean_curv + (1|subjectID)`).

The full results of this analysis, including effects per visual feature, and domain effects, can be found at https://rpubs.com/shariliu/nes_results in Section 5. Each of the visual features predicted the amplitude of univariate activity in at least one focal region. All of the VOE results in our focal regions (both positive and negative) held taking into account these features:

- Left and right SMG did not show a main effect of event (LSMG: [-0.076, 0.209], B =0.067, p = 0.361, two-tailed; RSMG: [-0.045, 0.252], B =0.103, p = 0.175, two-tailed), but both regions showed an interaction between event and domain, with a greater VOE effect for physical events (LSMG: [0.063, 0.347], B =0.205, p = 0.005, two-tailed; RSMG: [0.033, 0.328], B =0.18, p = 0.018, two-tailed).
- Neither left nor right STS showed a main effect of event (LSTS: [-0.103, 0.257], B =0.077, p = 0.403, two-tailed; RSTS: [-0.041, 0.247], B =0.103, p = 0.165, two-tailed).
- Neither bilateral V1 and bilateral MT showed a main effect of event (V1: [-0.137, 0.26], B =0.061, p = 0.547, two-tailed; MT: [-0.07, 0.163], B =0.047, p = 0.437, two-tailed).
- Both bilateral APC and RFC showed a main effect of event, responding more to unexpected than expected events (APC: [0.084, 0.422], B =0.253, p = 0.004, two-tailed; RFC: [0.084, 0.423], B =0.254, p = 0.004, two-tailed).

The domain effects from all domain-specific regions held, though the SMG domain responses were weaker after controlling for visual features:

- Left and right SMG responded more to physical events (LSMG: [0.024, 0.512], B = 0.268, p = 0.032, two-tailed; RSMG [0.04, 0.548], B = 0.294, p = 0.024, two-tailed)
- Left and right STS responded more to psychological events (LSTS [-0.705, -0.086], B = -0.396, p = 0.013, two-tailed; RSTS [-0.64, -0.139], B = -0.389, p = 0.003, two-tailed)

The domain effects from MD and early visual regions were no longer statistically significant, after controlling for all visual features, except for V1.

- Neither RFC nor APC showed a main effect of domain (APC: [-0.156, 0.426], B = 0.135, p = 0.365, two-tailed; RFC: [-0.006, 0.577], B = 0.286, p = 0.056, two-tailed)
- MT did not show a main effect of domain (MT: [-0.01, 0.389], B = 0.19, p = 0.064, two-tailed)
- V1 still responded more to physical than psychological events ([0.122, 0.813], B = 0.468, p = 0.008, two-tailed).

6.6 Results from originally selected MD ROIs

We originally pre-registered (1) bilateral insula and (2) right precentral/inferior frontal cortex as our focal MD ROIs. We discovered a mistake in this ROI definition analysis and, after fixing it, followed the same pre-registered procedure for selecting the two MD ROIs that appear in the main text. We report the results from these original two ROIs below for full transparency. See https://rpubs.com/shariliu/nes_results, Section 5, for full results.

In Experiment 1, neither bilateral IFC, nor bilateral insula, responded significantly more to unexpected than expected events (IFC: [-0.002, 0.265], B = 0.131, p = 0.056, two-tailed, BF = 0.065; insula: [-0.003, 0.167], B = 0.082, p = 0.06, two-tailed, BF = 0.039).

In Experiment 2, bilateral IFC, but not bilateral insula, responded significantly more to unexpected than expected events (IFC: [0.046, 0.383], B = 0.215, p = 0.013, two-tailed, BF = 0.297; insula: [-0.042, 0.163], B = 0.06, p = 0.253, two-tailed, BF = 0.016).

6.7 Responses to visual novelty in early visual regions

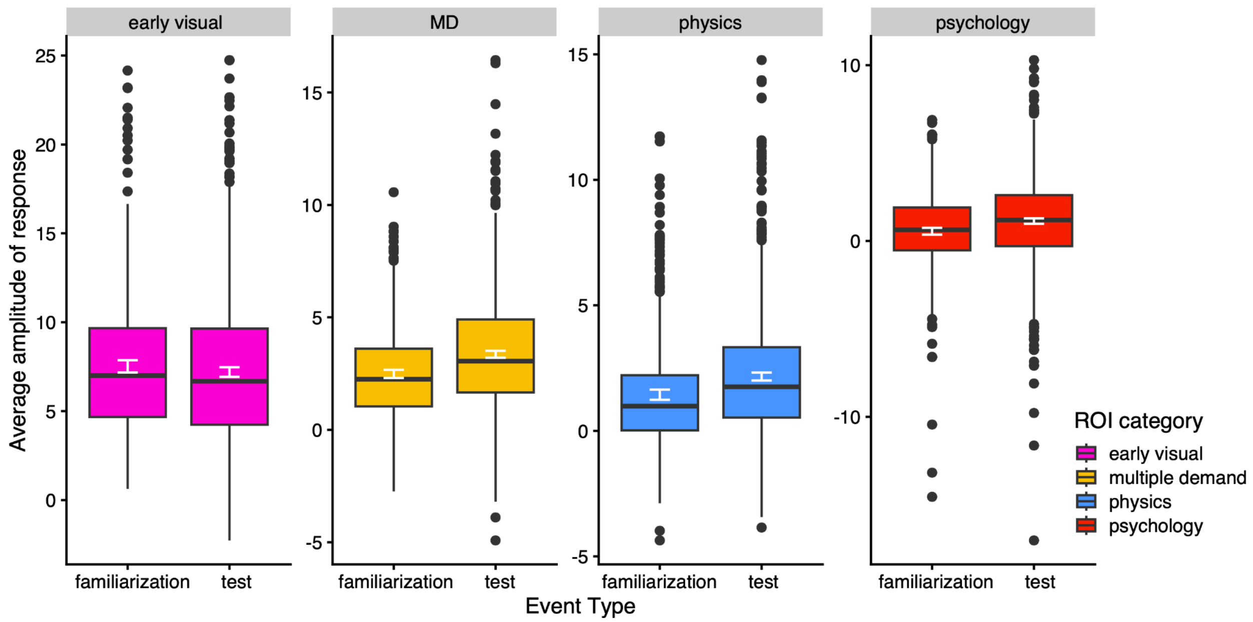

Figure S7. Average responses in focal ROIs to familiarization and test events, from psychology-action and physics in runs 1-2, matching the data for the confirmatory analyses. Test events include both expected and unexpected events. We found that whereas early visual regions responded more to visually new events (familiarization > test), all other regions responded more to conceptually new events (test > familiarization). Error bars indicate boostrapped 95% confidence intervals.

As one benchmark of whether our fROI analysis could detect visual novelty in our stimuli we compared the response to all familiarization events (more visually new) > all test events (less visually new) in our early visual ROIs from Experiment 2. We found stronger responses to more novel stimuli, in both bilateral V1 (B = 0.176, [0.006, 0.345], p=.043, two-tailed) and bilateral MT (B = 0.139, [0.044, 0.233], p=.004, two-tailed). We observed this effect, even though each new familiarization event is different from the test events in different ways in terms of the visual details. This confirms that it is possible to measure univariate responses broad fROIs to visual novelty defined over varying features across trials.

We also found that the early visual ROIs were the only focal regions to show greater responses to familiarization than test events. We found an ROI category by event interaction (likelihood ratio test, $p < .001$), such that the physics, psychology, and multiple demand ROIs all responded more to the conceptually relevant test events, more so than the visually new familiarization events (physics ROIs: B=-0.729, $p < .001$; psychology ROIs: B=-0.590, $p < .001$; MD ROIs: B = -0.853, $p < .001$, all two-tailed), in contrast to the early visual ROIs, which responded more to the visually novel familiarization events (B=0.314, p=.020). These results show that it is possible to measure a visual novelty effect in these early visual ROIs, which makes it more plausible that we could have measured a VOE effect if one existed in these regions.

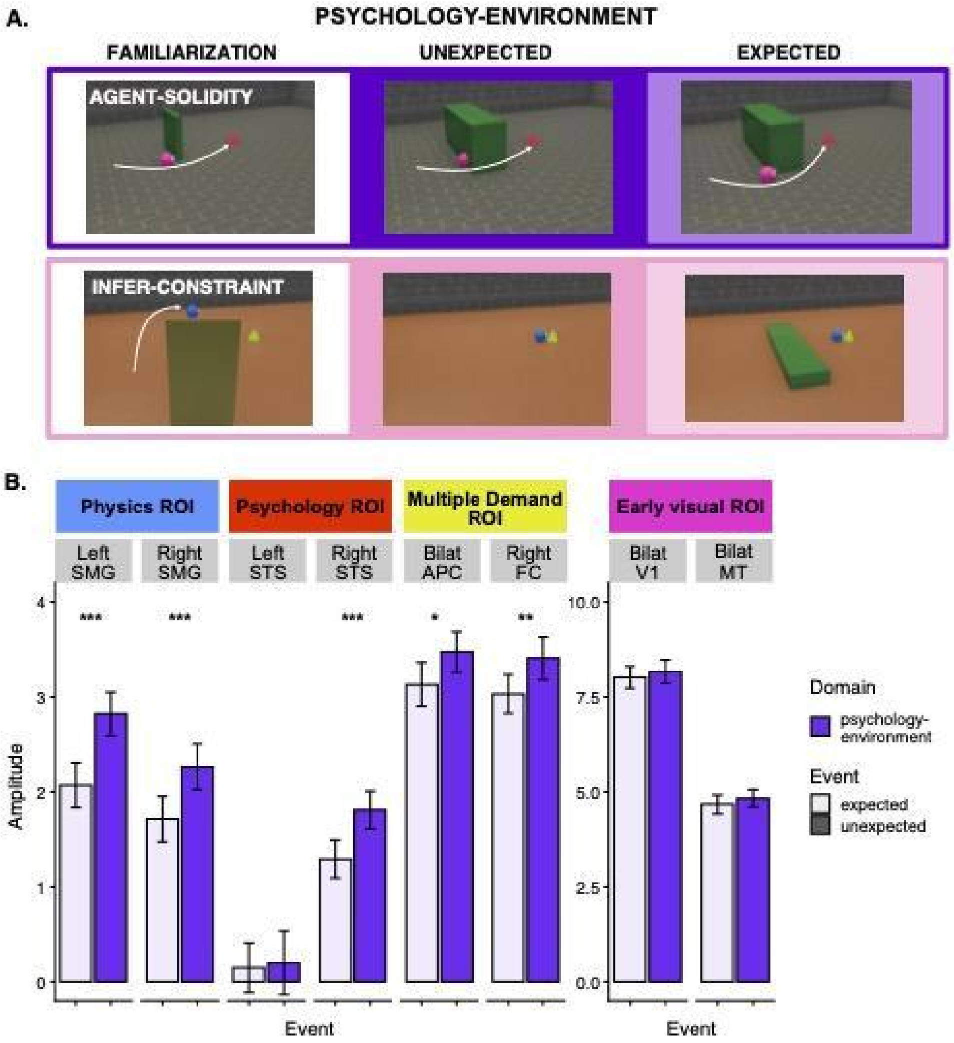

Figure S8. (A) Stimuli from the domain of intuitive psychology, wherein the actions of an agent lead to a surprising physical outcome in the surrounding environment (psychology-environment). In *agent-solidity*, an agent passes through a solid wall; in *infer-constraint*, an obstacle that explains an agent's action is missing. (B) Univariate responses towards these events in all focal regions, across all four runs. Error bars indicate within-subjects standard error.

6.8 VOE effects for psychology-environment events, all runs

Taking data from all 4 runs, we found that many non-focal regions responded significantly more to unexpected than expected psychology-environment events. See https://rpubs.com/shariliu/nes_results, Section 5, for results from all regions.
The domain-general regions:

- Left insula: [0.048, 0.167], B = 0.107, $p < .001$, two-tailed, BF = 1.509
- Right superior frontal [0.074, 0.278], B = 0.176, $p = 0.001$, two-tailed, BF = 1.648

The domain-specific regions:

- Right precentral/superior frontal [0.067, 0.215], B = 0.141, $p < .001$, two-tailed, BF = 3.974

- Right superior inferior frontal [0.167, 0.353], B = 0.26, $p < .001$, two-tailed, BF > 1000
- Left superior parietal [0.134, 0.472], B = 0.303, $p < .001$, two-tailed, BF = 4.051
- Right superior parietal [0.157, 0.475], B = 0.316, $p < .001$, two-tailed, BF = 15.888
- Left lateral and ventral visual [0.076, 0.275], B = 0.175, $p = 0.001$, two-tailed, BF = 1.933
- Right lateral and ventral visual [0.127, 0.384], B = 0.256, $p < .001$, two-tailed, BF = 12.163

6.9 VOE effects by task

We explored whether the VOE effect varied by task (e.g. permanence vs solidity), beyond by domain (e.g. physics vs psychology). We fit a mixed effects model on responses per scenario per task per ROI, extracted the coefficient and standard errors from the model, and plotted them in Figures S7-8. Because each slice of this data is small, we do not strongly interpret these results. However, qualitatively, we see that the tasks, across experiments, with the lowest neural VOE effect overall, across regions, are the permanence and infer-constraint tasks (Exp 2), and the efficiency task (Exp 1). By contrast, qualitatively, the task that evoked the highest responses across all regions was the agent-solidity task (Exp 2).

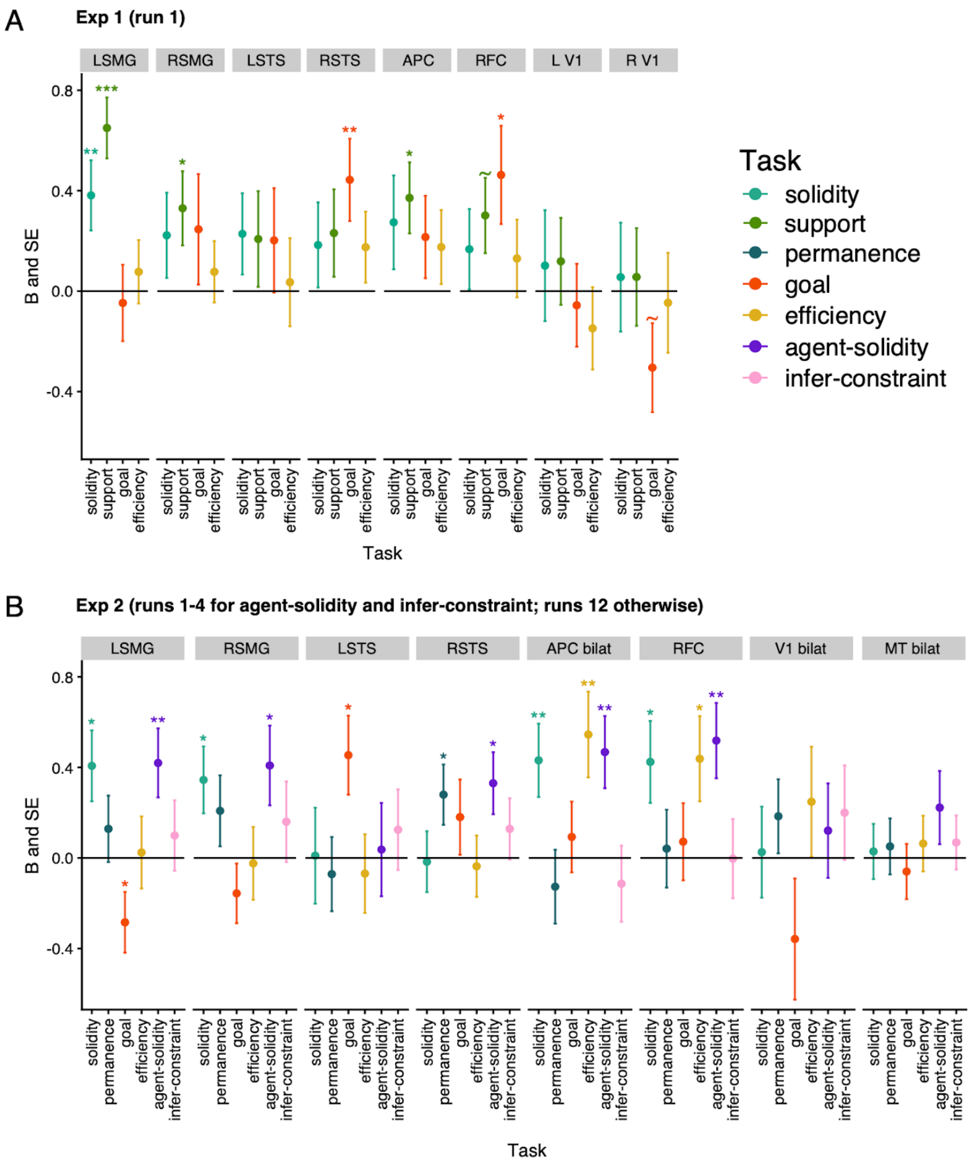

Figure S9. Per-task, per-region VOE effects for Experiments 1 and 2, organized by region. Error bars indicate the standard error of the B coefficient. ~ p < .10, * p < .05, ** p < .01, ***p < .001, two-tailed. This parallels Figure S8, except that Figure S8 is organized by task.

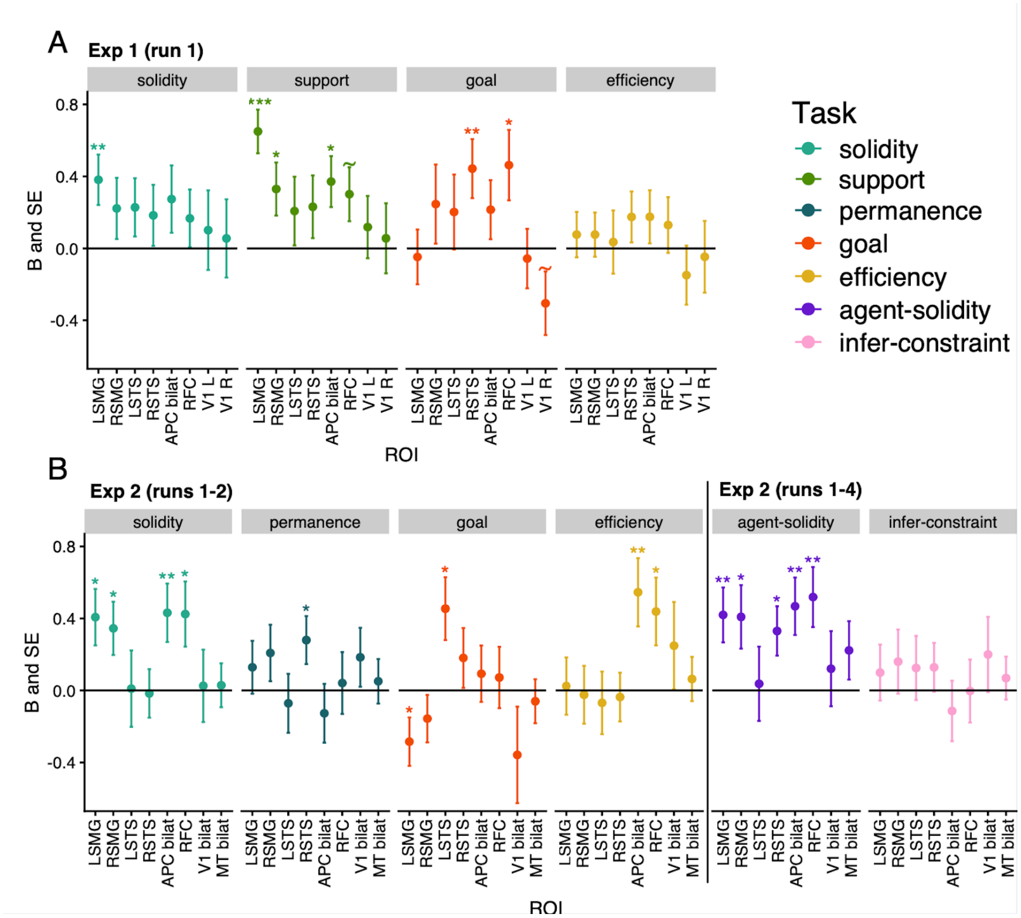

Figure S10. Estimate of neural VOE effect (positive values indicate greater responses to unexpected than expected) for each task across all focal regions for Experiments 1-2. Error bars indicate the standard error of the B coefficient. ~ p < .10, * p < .05, ** p < .01, ***p < .001, two-tailed. This parallels Figure S7, except that Figure S7 is organized by region.

7. Supplemental MVPA results

For both Experiments 1 and 2, we calculated the Euclidean distance for each participant for each region along the following category boundaries: events across domains, domains across events, events within domains (i.e. information about event within psychology-action and physics separately), and domains within events (i.e. information about domains within unexpected and expected events separately). To evaluate whether a given region had multivariate information about a given category boundary, we first computed the within vs between category distance for each boundary. Then we tested whether the within-category distances were significantly less than the between-category distances using a one-tailed Wilcoxon signed rank test. Below we will highlight the results, from Experiment 2, most relevant to our realization that we could not use MVPA to study the VOE effect from this work.

7.1 Robust univariate, and absent multivariate, event effects

In Experiment 2, both APC and RFC showed a univariate main effect of event. We planned to test for multivariate information about events that generalized across domains. However, neither of these regions contained multivariate information about event within domains (physics: $V = 257$, $p = 0.555$, one-tailed, $r = 0.104$; psychology: $V = 227$, $p = 0.756$, one-tailed, $r = 0.055$). Thus, it did not make sense to us to strongly interpret the null MVPA effect across domains (bilateral APC: $V = 273$, $p = 0.438$, one-tailed, $r = 0.137$; right frontal cortex: $V = 219$, $p = 0.8$, one-tailed, $r = 0.045$).

This stood in contrast to the robust domain multivariate effects. In Experiment 2, the regions that showed univariate domain effects, and no domain x event interaction, showed multivariate domain effects across event types:

- Bilateral V1: $V = 495$, $p < .001$, one-tailed, $r = 0.875$
- Bilateral MT: $V = 518$, $p < .001$, one-tailed, $r = 1.013$
- Bilateral APC: $V = 474$, $p < .001$, one-tailed, $r = 0.776$
- Left STS: $V = 378$, $p = 0.016$, one-tailed, $r = 0.425$
- Right STS: $V = 386$, $p = 0.011$, one-tailed, $r = 0.451$
- Right SMG: $V = 435$, $p < .001$, one-tailed, $r = 0.62$

The remaining focal region, left SMG, did not contain multivariate information about domains across events, $V = 314$, $p = 0.18$, one-tailed, $r = 0.237$. Instead, its domain boundary was marginally significant for unexpected events $V = 345$, $p = 0.067$, one-tailed, $r = 0.324$, and not significant for expected events, $V = 310$, $p = 0.200$, one-tailed, $r = 0.227$

See https://rpubs.com/shariliu/nes_results, Section 5, for full MVPA results for all regions, for all event boundaries, and for both experiments.

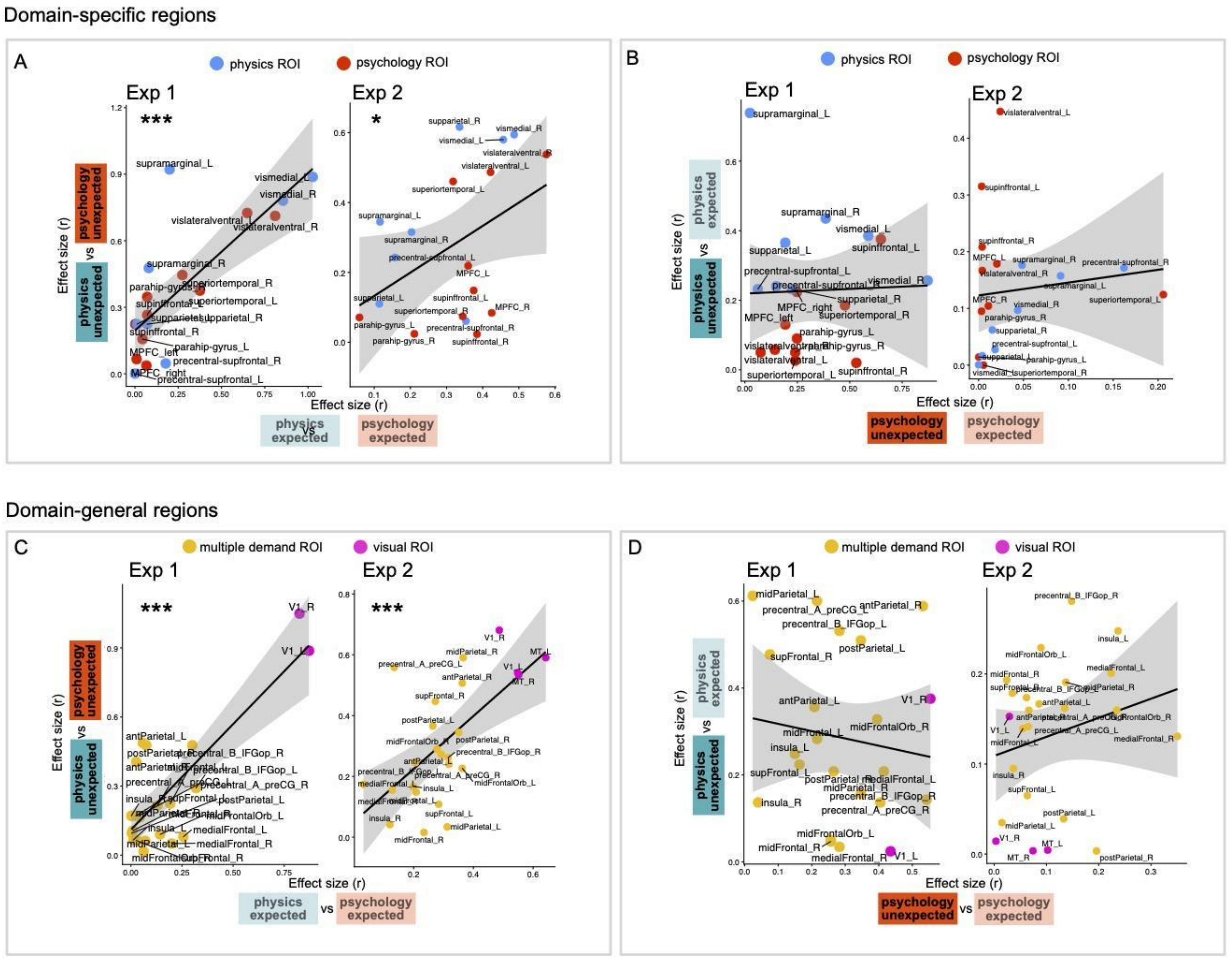

Figure S11. Multivariate effect size results across all domain-specific regions (A-B) and domain-general regions (C-D) from the exploratory results of Experiment 1, and the confirmatory results of Experiment 2. (A) and (C) show correspondence between event information across domains. (B) and (D) show correspondence between domain information across events. We found that the responses in both categories of regions were organized by domain, and not by event. ~ $p < .10$, * $p < .05$, ** $p < .01$, ***$p < .001$, one-tailed, non-parametric test for independence.

7.2 Multivariate region-by-region analysis

Here we report the results from a pre-registered confirmatory univariate analysis, studying the organization of information about domains and events across a large set of regions. However, after finding that regions that show a univariate VOE effect do not show a multivariate effect of event, within or across domains, we have decided to move that analysis, and this analysis, to the SM. Given that the multivariate information about domains is much stronger than multivariate information about events in our focal regions, we do not strongly interpret the following results, which show that both domain-specific and domain-general regions are organized more so by domain than by event. However, an alternative interpretation of these results is that the VOE response is encoded in the amplitude of activity voxels within an ROI, but the spatial pattern of this response is not reliable. Below we report them for full transparency.

For each region, we computed the effect size for the multivariate category boundary for events across and within domains, and information about domains across and within events. In Exp 1 and 2, we conducted this analysis on the 18 domain-specific regions, and on the 24 domain-general regions (22 for Experiment 1; minus left and right MT, which we had no way to define). The voxel selection procedure was identical to the univariate analyses, except that we selected the top 100 voxels from each region in each hemisphere, to maximize the number of regions available as input. In this analysis, we focused on domain-within-events and events-within-domain effect sizes - that is, how much information there is about a given category boundary in a region, relative to variance and sample size. For each region, we computed a pair of MVPA effect sizes, *r_event_psychology* and *r_event_physics*, that describes the amount of event information for each domain separately. For each region, we also computed a second pair of MVPA effect sizes, *r_domain_expected* and *r_domain_unexpected*, that describes the amount of domain information for each event type separately.

The main question is whether domain-specific regions and domain-general regions are organized primarily by domain and event, respectively. We found in Exp 1, and hypothesized and found in Exp 2, that patterns of activity across domain-specific regions and domain-general regions will be organized more by domain than by event. To test this hypothesis, we calculated a correlation value, using a nonparametric test of independence, which uses permutation to test the null hypothesis that two vectors are statistically independent, but making no assumption about the linearity of their dependence. For each set of regions, we calculated a correlation value relating information about events across domains, across regions (*r_event* = cor(*r_event_psychology*, *r_event_physics*), and a second correlation value relating information about domains across events, across regions (*r_domain* = cor(*r_domain_expected, r_domain_unexpected)*).

In Exp 2 we predicted that for both domain-specific and domain-general regions, (1) *r_domain* will be significantly larger than expected by chance (one-tailed prediction), and (2) *r_domain* will be larger than *r_event* (one-tailed prediction). To test this prediction, we used bootstrapping to compute the difference between these two values under the null hypothesis (4000 iterations). We calculated a p-value by counting the number of permuted differences out of the 4000 that was equal to or greater than the observed difference between *r_domain* and *r_event*. Our significance threshold was $p = .05$, one-tailed.

Exploratory results from Experiment 1 showed that for both domain-specific and domain-general regions, the degree to which a region contained information to distinguish psychological expected vs unexpected events did not significantly predict that same region's information to distinguish between physical expected and unexpected events (domain-specific: cor = -0.138, $p = 0.740$; domain-general: cor = -0.138, $p = 0.740$). In contrast, the degree to which a region contained information to distinguish between expected psychological vs physical events strongly predicted the degree to which that region distinguishes between unexpected psychological vs physical events (domain-specific: cor = 0.783, $p <.001$; domain-general: cor = 0.788, $p < .001$). Comparing the two correlations against each other using bootstrapping to generate the distribution of correlations expected under the null hypothesis (4000

iterations), we found that the domain correlation was stronger than the event correlation for domain-specific regions (95% CI [0.269, 1.065], $p = 0.002$), and for domain-general regions (95% CI [0.011, 1.407], $p = 0.048$). We then pre-registered these predictions in Experiment 2. The confirmatory analyses of Experiment 2 converged with these findings. There was no significant relationship between event information across psychological and physical events in either domain-specific regions (cor = 0.225, $p = 0.148$) or domain-general regions (cor = 0.225, $p = 0.146$). There was a correspondence between domain information across event types in both domain-specific regions (cor = 0.439, $p = 0.036$) and domain-general regions (cor = 0.637, $p < .001$). However, the two correlations were not significantly different from each other in either domain-specific (95% CI [-0.232, 0.642], $p = 0.229$), nor domain-general regions (95% CI [-0.031, 0.821], $p = 0.064$). Taking these results literally, for both putatively domain-general and domain-specific regions, multivariate information across regions is organized by domain, and not by event. However, for the reasons described in the first paragraph of this section, we do not strongly interpret these results.

8. Whole-brain group analyses

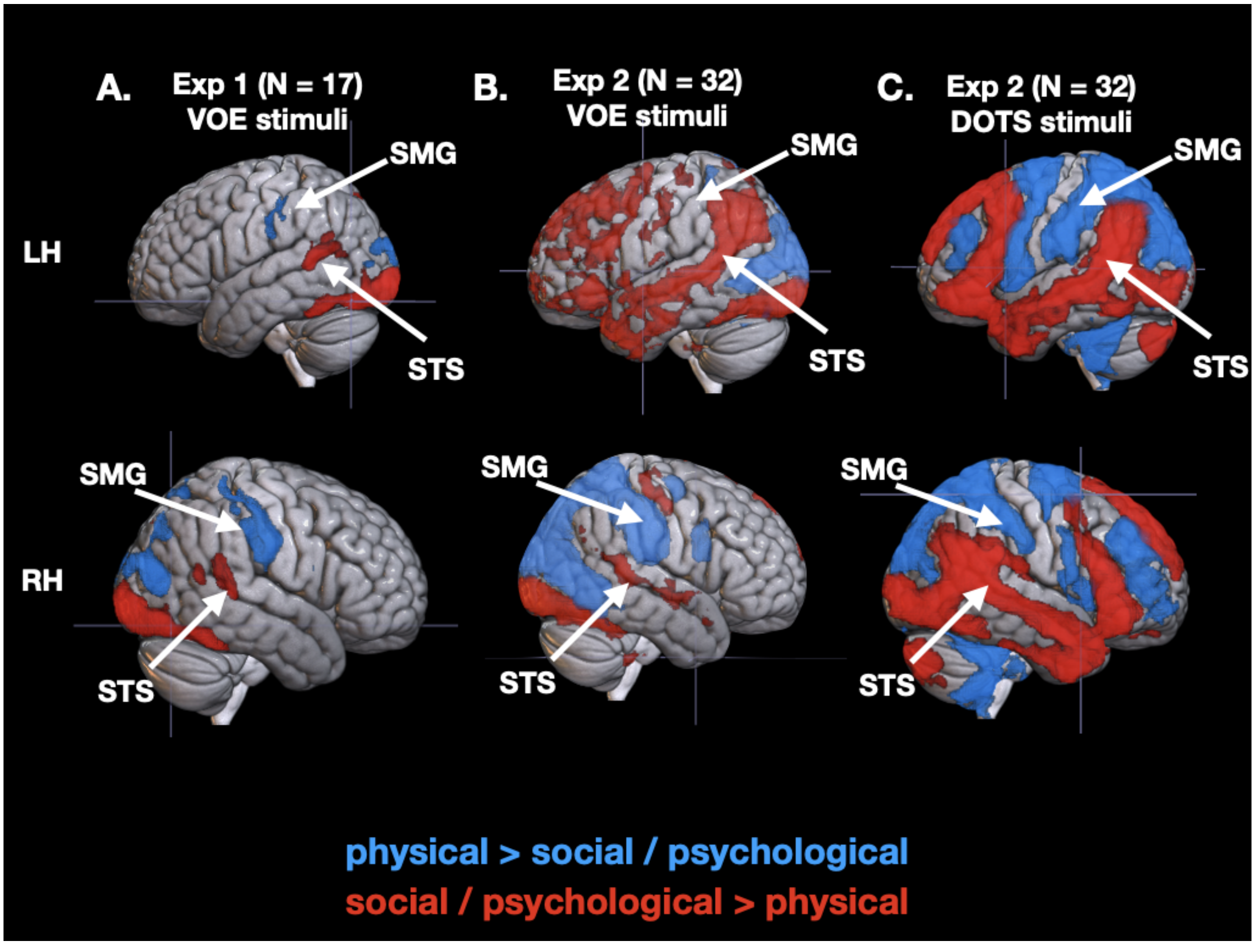

Figure S12. Results from whole-brain random effects analyses from the social/psychological vs physical contrast for the VOE task in Experiments 1-2 (A-B), and the DOTS task in Experiment 2 (C), generated from a non-parametric one-tailed test using FSL's `randomise()` and 5000 iterations, at a threshold of $p < .05$, TCFE. We additionally applied variance smoothing over the data for Experiment 1 (σ=6mm) following the recommendation of Nichols and Holmes (2002), due to the small sample size (< 20 people). Arrows point to the focal physics and psychology regions of interest (SMG and STS). Abbreviations: LH = left hemisphere; RH = right hemisphere; SMG = supramarginal gyrus; STS = superior temporal sulcus.

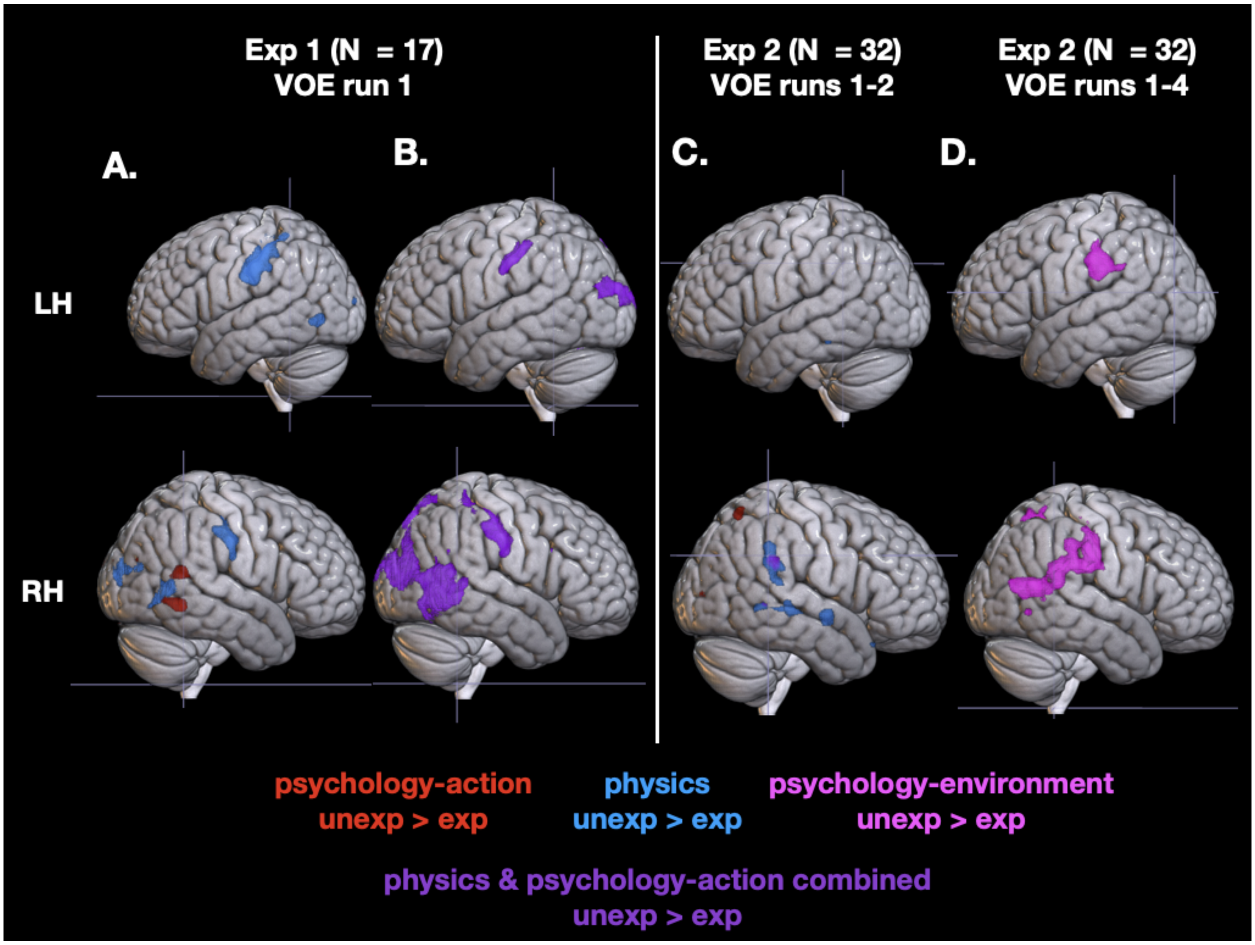

Figure S13. Group results on the neural VOE effect (unexpected > expected) from Experiment 1 (A-B) and Experiment (2) (C-D). Purple regions in (B) and (C) indicate the VOE effect folding over psychology-action and physics events, and pink regions in (D) indicate the VOE effect over psychology-environment events. All maps generated from a non-parametric one-tailed test using FSL's `randomise()` and 5000 iterations, at a threshold of $p < .05$, TCFE, except for the VOE effects for psychology-action and physics events in panel (C), which were absent at this threshold, and shown at a more lenient threshold of $p < .20$, TCFE. We additionally applied variance smoothing over the data for Experiment 1 (σ=6mm) following the recommendation of Nichols and Holmes (2002), due to the sample size (< 20 people). Abbreviations: LH = left hemisphere; RH = right hemisphere.

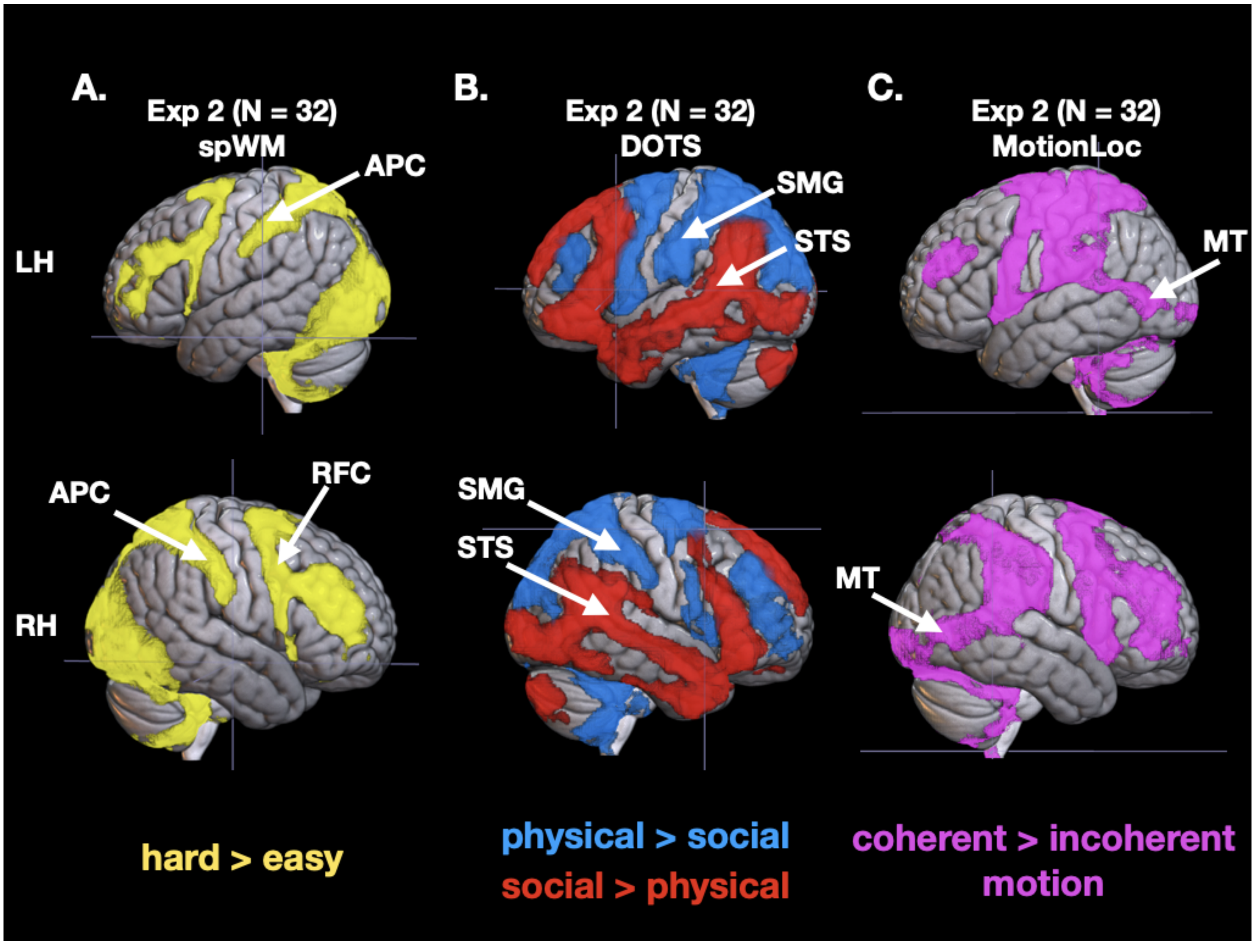

Figure S14. Group results over localizer tasks in Experiment 2. (A) Hard > easy contrast from the MD localizer. (B) Physical vs social contrast from the DOTS localizer. (C) Coherent > incoherent motion from MT localizer. These maps were generated from a non-parametric one-tailed test using FSL's `randomise()` and 5000 iterations, at a threshold of $p < .05$, TCFE. Arrows point to the focal physics and psychology regions of interest (SMG and STS), the focal MD regions of interest (APC and RFC), and one of the two focal early visual regions of interest (MT). Abbreviations: LH = left hemisphere; RH = right hemisphere; APC = anterior parietal cortex; RFC = right frontal cortex; SMG = supramarginal gyrus; STS = superior temporal sulcus; MT = motion-sensitive area.

9. References

Abraham, Alexandre, Fabian Pedregosa, Michael Eickenberg, Philippe Gervais, Andreas Mueller, Jean Kossaifi, Alexandre Gramfort, Bertrand Thirion, and Gael Varoquaux. 2014. “Machine Learning for Neuroimaging with Scikit-Learn.” *Frontiers in Neuroinformatics* 8. https://doi.org/10.3389/fninf.2014.00014.

Avants, B.B., C.L. Epstein, M. Grossman, and J.C. Gee. 2008. “Symmetric Diffeomorphic Image Registration with Cross-Correlation: Evaluating Automated Labeling of Elderly and Neurodegenerative Brain.” *Medical Image Analysis* 12 (1): 26–41. https://doi.org/10.1016/j.media.2007.06.004.

Bates, Douglas, Martin Mächler, Ben Bolker, and Steve Walker. "Fitting Linear Mixed-Effects Models Using lme4." *Journal of Statistical Software 67* (2015): 1-48.

Behzadi, Yashar, Khaled Restom, Joy Liau, and Thomas T. Liu. 2007. “A Component Based Noise Correction Method (CompCor) for BOLD and Perfusion Based fMRI.” *NeuroImage* 37 (1): 90–101. https://doi.org/10.1016/j.neuroimage.2007.04.042.

Bennett, Craig M., and Michael B. Miller. 2010. “How Reliable Are the Results from Functional Magnetic Resonance Imaging?” Annals of the New York Academy of Sciences 1191 (1): 133–55. https://doi.org/10.1111/j.1749-6632.2010.05446.x.

Dale, Anders M., Bruce Fischl, and Martin I. Sereno. 1999. “Cortical Surface-Based Analysis: I. Segmentation and Surface Reconstruction.” *NeuroImage* 9 (2): 179–94. https://doi.org/10.1006/nimg.1998.0395.

Esteban, Oscar, Ross Blair, Christopher J. Markiewicz, Shoshana L. Berleant, Craig Moodie, Feilong Ma, Ayse Ilkay Isik, et al. 2018. “FMRIPrep.” *Software*. Zenodo. https://doi.org/10.5281/zenodo.852659.

Esteban, Oscar, Christopher Markiewicz, Ross W Blair, Craig Moodie, Ayse Ilkay Isik, Asier Erramuzpe Aliaga, James Kent, et al. 2018. “fMRIPrep: A Robust Preprocessing Pipeline for Functional MRI.” *Nature Methods*. https://doi.org/10.1038/s41592-018-0235-4.

Evans, AC, AL Janke, DL Collins, and S Baillet. 2012. “Brain Templates and Atlases.” *NeuroImage* 62 (2): 911–22. https://doi.org/10.1016/j.neuroimage.2012.01.024.

Fedorenko, Evelina, John Duncan, and Nancy Kanwisher. 2013. “Broad Domain Generality in Focal Regions of Frontal and Parietal Cortex.” Proceedings of the National Academy of Sciences of the United States of America 110 (41): 16616–21.

Fonov, VS, AC Evans, RC McKinstry, CR Almli, and DL Collins. 2009. “Unbiased Nonlinear Average Age-Appropriate Brain Templates from Birth to Adulthood.” *NeuroImage*, Organization for human brain mapping 2009 annual meeting, 47, Supplement 1: S102. https://doi.org/10.1016/S1053-8119(09)70884-5.

Fouragnan, Elsa, Chris Retzler, and Marios G. Philiastides. "Separate neural representations of prediction error valence and surprise: Evidence from an fMRI meta‐analysis." *Human brain mapping 39*, no. 7 (2018): 2887-2906.

Glasser, Matthew F., Stamatios N. Sotiropoulos, J. Anthony Wilson, Timothy S. Coalson, Bruce Fischl, Jesper L. Andersson, Junqian Xu, et al. 2013. “The Minimal Preprocessing Pipelines for the Human Connectome Project.” *NeuroImage*, Mapping the connectome, 80: 105–24. https://doi.org/10.1016/j.neuroimage.2013.04.127.

Gorgolewski, K., C. D. Burns, C. Madison, D. Clark, Y. O. Halchenko, M. L. Waskom, and S. Ghosh. 2011. “Nipype: A Flexible, Lightweight and Extensible

Neuroimaging Data Processing Framework in Python." *Frontiers in Neuroinformatics* 5: 13. https://doi.org/10.3389/fninf.2011.00013.

Gorgolewski, Krzysztof J., Oscar Esteban, Christopher J. Markiewicz, Erik Ziegler, David Gage Ellis, Michael Philipp Notter, Dorota Jarecka, et al. 2018. "Nipype." *Software*. Zenodo. https://doi.org/10.5281/zenodo.596855.

Greve, Douglas N, and Bruce Fischl. 2009. "Accurate and Robust Brain Image Alignment Using Boundary-Based Registration." *NeuroImage* 48 (1): 63–72. https://doi.org/10.1016/j.neuroimage.2009.06.060.

Jenkinson, Mark, Peter Bannister, Michael Brady, and Stephen Smith. 2002. "Improved Optimization for the Robust and Accurate Linear Registration and Motion Correction of Brain Images." *NeuroImage* 17 (2): 825–41. https://doi.org/10.1006/nimg.2002.1132.

Kang, Min Jeong, Ming Hsu, Ian M. Krajbich, George Loewenstein, Samuel M. McClure, Joseph Tao-yi Wang, and Colin F. Camerer. "The wick in the candle of learning: Epistemic curiosity activates reward circuitry and enhances memory." *Psychological science 20*, no. 8 (2009): 963-973.

Klein, Arno, Satrajit S. Ghosh, Forrest S. Bao, Joachim Giard, Yrjö Häme, Eliezer Stavsky, Noah Lee, et al. 2017. "Mindboggling Morphometry of Human Brains." *PLOS Computational Biology* 13 (2): e1005350. https://doi.org/10.1371/journal.pcbi.1005350.

Kuznetsova, Alexandra, Per B. Brockhoff, and Rune HB Christensen. "lmerTest package: tests in linear mixed effects models." *Journal of statistical software 82* (2017): 1-26.

Kosakowski, Heather L., Michael A. Cohen, Atsushi Takahashi, Boris Keil, Nancy Kanwisher, and Rebecca Saxe. 2022. "Selective Responses to Faces, Scenes, and Bodies in the Ventral Visual Pathway of Infants." *Current Biology: CB* 32 (2): 265-274.e5. https://doi.org/10.1016/j.cub.2021.10.064.

Lanczos, C. 1964. "Evaluation of Noisy Data." *Journal of the Society for Industrial and Applied Mathematics Series B Numerical Analysis* 1 (1): 76–85. https://doi.org/10.1137/0701007.

Lenth, Russell V. "Least-squares means: the R package lsmeans." *Journal of statistical software 69* (2016): 1-33.

Nishimoto, Shinji, An T. Vu, Thomas Naselaris, Yuval Benjamini, Bin Yu, and Jack L. Gallant. 2011. "Reconstructing Visual Experiences from Brain Activity Evoked by Natural Movies." *Current Biology: CB* 21 (19): 1641–46. https://doi.org/10.1016/j.cub.2011.08.031.

Parris, Ben A., Gustav Kuhn, Guy A. Mizon, Abdelmalek Benattayallah, and Tim L. Hodgson. "Imaging the impossible: An fMRI study of impossible causal relationships in magic tricks." *Neuroimage 45*, no. 3 (2009): 1033-1039.

Patriat, Rémi, Richard C. Reynolds, and Rasmus M. Birn. 2017. "An Improved Model of Motion-Related Signal Changes in fMRI." *NeuroImage* 144, Part A (January): 74–82. https://doi.org/10.1016/j.neuroimage.2016.08.051.

Pramod, R. T., Michael A. Cohen, Joshua B. Tenenbaum, and Nancy Kanwisher. 2022. "Invariant Representation of Physical Stability in the Human Brain." *eLife* 11 (May). https://doi.org/10.7554/eLife.71736.

Power, Jonathan D., Anish Mitra, Timothy O. Laumann, Abraham Z. Snyder, Bradley L. Schlaggar, and Steven E. Petersen. 2014. "Methods to Detect, Characterize, and Remove Motion Artifact in Resting State fMRI." *NeuroImage* 84 (Supplement C): 320–41. https://doi.org/10.1016/j.neuroimage.2013.08.048.

Pruim, Raimon H. R., Maarten Mennes, Daan van Rooij, Alberto Llera, Jan K. Buitelaar, and Christian F. Beckmann. 2015. “ICA-AROMA: A Robust ICA-Based Strategy for Removing Motion Artifacts from fMRI Data.” *NeuroImage* 112 (Supplement C): 267–77. https://doi.org/10.1016/j.neuroimage.2015.02.064.

Rajimehr, R., Devaney, K. J., Bilenko, N. Y., Young, J. C., & Tootell, R. B. H. (2011). The “parahippocampal place area” responds preferentially to high spatial frequencies in humans and monkeys. PLoS Biology, 9(4), e1000608. https://doi.org/10.1371/journal.pbio.1000608

Satterthwaite, Theodore D., Mark A. Elliott, Raphael T. Gerraty, Kosha Ruparel, James Loughead, Monica E. Calkins, Simon B. Eickhoff, et al. 2013. “An improved framework for confound regression and filtering for control of motion artifact in the preprocessing of resting-state functional connectivity data.” *NeuroImage* 64 (1): 240–56. https://doi.org/10.1016/j.neuroimage.2012.08.052.

Shu, Tianmin, Abhishek Bhandwaldar, Chuang Gan, Kevin Smith, Shari Liu, Dan Gutfreund, Elizabeth Spelke, Joshua Tenenbaum, and Tomer Ullman. 18--24 Jul 2021. “AGENT: A Benchmark for Core Psychological Reasoning.” In Proceedings of the 38th International Conference on Machine Learning, edited by Marina Meila and Tong Zhang, 139:9614–25. Proceedings of Machine Learning Research. PMLR.

Smith, Kevin, Lingjie Mei, Shunyu Yao, Jiajun Wu, Elizabeth Spelke, Josh Tenenbaum, and Tomer Ullman. 2019. “Modeling Expectation Violation in Intuitive Physics with Coarse Probabilistic Object Representations.” Advances in Neural Information Processing Systems 32.

Tustison, N. J., B. B. Avants, P. A. Cook, Y. Zheng, A. Egan, P. A. Yushkevich, and J. C. Gee. 2010. “N4ITK: Improved N3 Bias Correction.” *IEEE Transactions on Medical Imaging* 29 (6): 1310–20. https://doi.org/10.1109/TMI.2010.2046908.

Zhang, Y., M. Brady, and S. Smith. 2001. “Segmentation of Brain MR Images Through a Hidden Markov Random Field Model and the Expectation-Maximization Algorithm.” *IEEE Transactions on Medical Imaging* 20 (1): 45–57. https://doi.org/10.1109/42.906424.
